# CO_2_, nitrogen deposition and a discontinuous climate response drive water use efficiency in global forests

**DOI:** 10.1038/s41467-021-25365-1

**Published:** 2021-08-31

**Authors:** Mark A. Adams, Thomas N. Buckley, Dan Binkley, Mathias Neumann, Tarryn L. Turnbull

**Affiliations:** 1 Department of Chemistry and Biotechnology, Faculty of Science, Engineering and Technology, Swinburne University of Technology, Melbourne, VIC Australia; 2 School of Life and Environmental Sciences, University of Sydney, Sydney, NSW Australia; 3 Department of Plant Sciences, College of Agricultural and Environmental Sciences, University of California, Davis, CA USA; 4 School of Forestry, Northern Arizona University, Flagstaff, AZ USA; 5 Institute of Silviculture, Department of Forest and Soil Sciences, University of Natural Resources and Life Sciences, Vienna, Austria

**Keywords:** Plant physiology, Carbon cycle, Hydrology, Atmospheric chemistry

## Abstract

Reduced stomatal conductance is a common plant response to rising atmospheric CO_2_ and increases water use efficiency (*W*). At the leaf-scale, *W* depends on water and nitrogen availability in addition to atmospheric CO_2_. In hydroclimate models *W* is a key driver of rainfall, droughts, and streamflow extremes. We used global climate data to derive Aridity Indices (AI) for forests over the period 1965–2015 and synthesised those with data for nitrogen deposition and *W* derived from stable isotopes in tree rings. AI and atmospheric CO_2_ account for most of the variance in *W* of trees across the globe, while cumulative nitrogen deposition has a significant effect only in regions without strong legacies of atmospheric pollution. The relation of aridity and *W* displays a clear discontinuity. *W* and AI are strongly related below a threshold value of AI ≈ 1 but are not related where AI > 1. Tree ring data emphasise that effective demarcation of water-limited from non-water-limited behaviour of stomata is critical to improving hydrological models that operate at regional to global scales.

## Abstract (web summary)

Water use efficiency is a key measure of plant responses to climate change. Here, the authors investigate its control by CO2, nitrogen deposition, and water availability using a global tree-ring dataset. They find an aridity threshold and quantify changes in control over the past 50 years.

## Introduction

Leaf stomata are critical controls of plant interactions with the atmosphere. Via their conductance (*g*_s_), stomata control the dominantly inward fluxes of CO_2_ to leaves and outward fluxes of water to the atmosphere. Intrinsic water use efficiency (defined as *W* (µmol mol^−1^) *=* *A/g*_s_, where *A* is the net CO_2_ assimilation rate and *g*_s_ is stomatal conductance to CO_2_), or the ratio of the flux of carbon to that of water (corrected for differences in vapour pressure deficit (VPD)), has mostly increased with the concentration of CO_2_ in the atmosphere (*c*_a_) for the past century^1–3^. In a recent synthesis of global data^4^, significant positive responses of *W* to *c*_a_ were recorded by 95% of all observations. However, the global rate of increase in *W* per unit atmospheric CO_2_ slowed markedly after the 1960s and approached zero after 2000^4^. The slowing rate of increase in *W* could be due to other factors that limit stomatal responses to CO_2_, including the availabilities of water and/or nitrogen.

Based on modelling, Terrer et al.^5^ concluded that for ~65% of the world’s vegetation, nitrogen availability exerted control of growth distinct from that exerted by *c*_a_. Similarly, limited nitrogen supplies constrained grassland responses to *c*_a_ under free-air CO_2_ enrichment (FACE) conditions^6^. Earlier FACE experiments with trees supported the notion of a CO_2_ fertilisation effect, but over the longer term, N limitations became apparent^7^. This conclusion is supported by recent meta-analyses^8,9^ which suggest that the rise in *c*_a_ may be responsible for long-term reductions in leaf N that are, in turn, acting as negative feedbacks to the CO_2_-fertilisation effect.

The unquestioned sensitivity of stomatal conductance to leaf dehydration—as a result of either atmospheric or soil water deficits—varies according to a trade-off between efficiency and safety^10,11^. At whole tree and ecosystem scales, the sensitivity and regulation of stomatal conductance are central to both growth and survival under drought conditions^12,13^. Via their stomata, plants control a sizeable proportion of all water returned to the atmosphere (e.g. refs. ^14,15^).

Missing from the literature, as far as we are aware, are comprehensive (addressing both spatial and temporal scales) analyses of individual and combined effects of water and nitrogen availability on *W*. Here, we use a multi-biome data set of isotope series derived from the rings of 411 trees (containing ~11,000 individual measures of annual *W*) spread across 349 sites, to address this gap in knowledge. We synthesise the data for *W* with climate data for each site using the CRU database^16^ and the TerraClimate database^17^—two of the most rigorous and widely used sources of climate data. We then add nitrogen deposition data for each site, as used by the Atmospheric Chemistry and Climate Model Intercomparison Project (ACCMIP)^18,19^. While N deposition cannot be directly equated with endogenous N availability, a recent review highlighted its role in altering N availability^20^. We use summed deposition over a 20-year period as it is the cumulative effect of N deposition—not the effect of a single year of input—which influences ecosystem properties. We hypothesise: (1) that *W* should vary inversely with water availability, independently of *c*_a_; (2) that inputs of N from the atmosphere should lead to increases in W, by helping mitigate *c*_a_-driven reductions in leaf N and *A*_max_. We find that *W* decreases linearly with Aridity Index (AI) in the world’s arid ecosystems, up to the point where rainfall and potential evaporation are more or less equal. Beyond that point (moist ecosystems), *W* shows little sensitivity to AI. Nitrogen deposition had positive effects on *W* in the eastern USA and central Europe during the first few decades of the study period (1965–1985). In recent decades (1995–2015) those effects have faded. Instead, nitrogen deposition now most enhances the positive effects of rising CO_2_ on *W* in low-moderate N deposition zones.

## Results

### Spatial patterns of tree ring, nitrogen and climate data

**Fig. 1:**
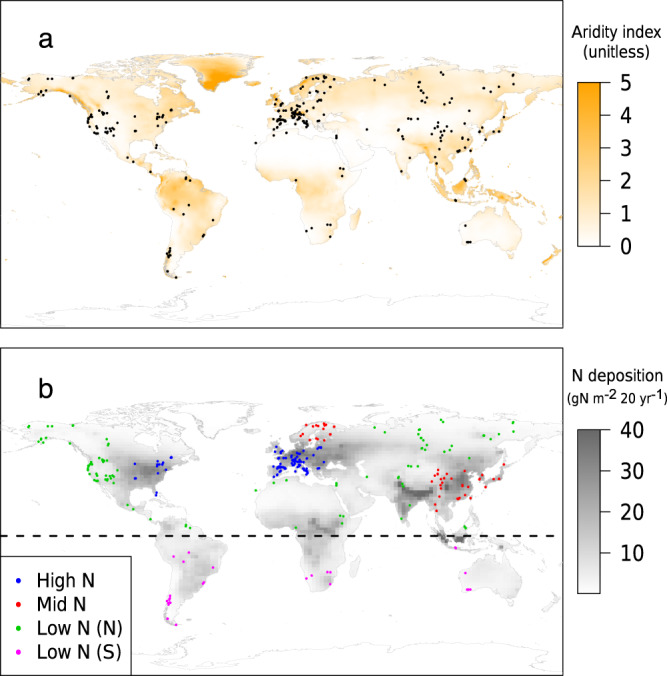
Geographic locations, climate and N deposition for 349 sites used in this study. **a** Aridity Index (AI, mm mm^−1^). **b** Annual N deposition (gN m^−2^ 20 years^−1^). All climate and N deposition data were extracted from published literature (see Methods). Maps for climate and N deposition are based on the year 2000. Tree ring study sites were allocated to nominal N deposition zones (see Methods).

Tree ring data for annual *W* (µmol mol^−1^) are unevenly distributed with respect to climate and nitrogen deposition (Fig. 1 and Supplementary Fig. 1). North America, Europe and to a lesser extent East Asia, are over-represented relative to other areas, and especially relative to the Southern Hemisphere. While extremes of both P and potential evapotranspiration (PET) (i.e. >3000 mm year^−1^) are more sparsely represented (Supplementary Fig. 1a, b), for AI there is a good spread of sites across the range from AI = 0 to 5 (Fig. 1a). Similarly, each geographically defined region of nitrogen (N) deposition (See Methods) is well populated (Fig. 1b).

### Temporal patterns of tree ring, nitrogen and climate data

N deposition differed significantly among sites (*p* < 0.05) within Köppen climatic zones (Supplementary Fig. 2a). For all Köppen zones, nitrogen deposition increased with increasing *c*_a_ and thus with time (Supplementary Fig. 2e). Known reductions in rates of N deposition in some geographic regions in recent years (see Methods) were not reflected in overall changes within Köppen zones. Mean P, PET and AI for study sites followed a broadly predictable pattern in relation to Köppen climate (Supplementary Fig. 2b–d). There were clear changes in PET and/or P with increasing *c*_a_ over the study period (Supplementary Fig. 2f–h). Notable were reductions in P for study sites within Köppen A climates (Supplementary Fig. 2f) and generally consistent increases in PET at study sites in all zones bar Köppen B (Supplementary Fig. 2g). Consequently, AI typically declined throughout the study period (site climates became drier; Supplementary Fig. 2h), with the exception of sites in Köppen B climates.

### Relations of *W* to aridity

**Fig. 2:**
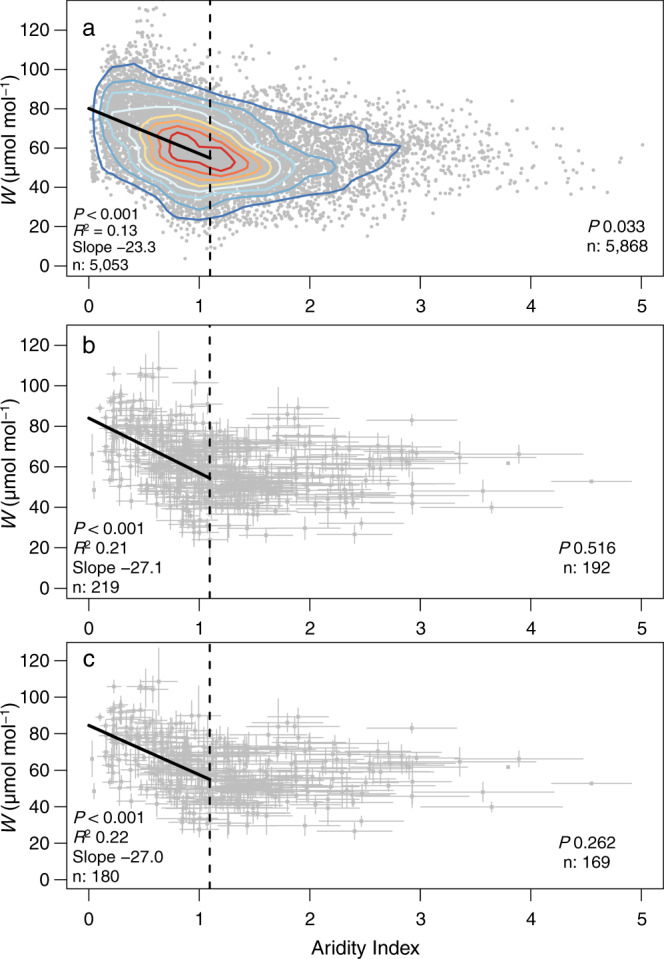
Relationship of intrinsic water use efficiency (*W*, µmol mol^−1^) to aridity index (precipitation/potential evapotranspiration, mm mm^−1^), 1965–2015. *P* values are from linear regressions using F-statistics. **a** All observations of *W*, as derived from individual tree rings. **b** Observations binned by sample tree. **c** Observations binned by study site. The breakpoint in AI is indicated by a broken line (see Methods for description of breakpoint analysis). In Panel **a**, the density of data points is indicated via lines demarking 10% increments in the total observations (from 20 to 90%).

When all observations for *W* were considered (Fig. 2a), or when data were grouped by a tree (Fig. 2b) or by the site (Fig. 2c), there was a clear discontinuity or threshold in the relationship of *W* to AI at AI ≈ 1. Excluding extreme observations (32 observations where AI > 5) had no effect on statistical analysis. Annual *W* was highly significantly (*p* < 0.001) and negatively correlated with AI < 1, but unrelated when AI > 1. It is worth noting that a clear majority of sites providing data for AI > 1 are less geographically dispersed (e.g. Europe and the northeast USA) than sites providing data for AI < 1 (which are more widespread geographically). For AI > 1, annual *W* was distributed around a mean of 55 µmol mol^−1^ (Fig. 2). Both Angiosperms and Gymnosperms contributed to the discontinuous dependence of *W* on AI (Supplementary Fig. 3), with Gymnosperms typically showing greater *W* than Angiosperms. When binned by AI class (Table 1), mean and median values (1965–2015) for *W* were highly consistent. *W* was invariant for AI > 1 but increased strongly as AI declined from 1.0 to 0.0.

**Table 1:** Summary statistics for annual *W* (µmol mol^−1^) derived from tree rings across aridity classes.

Aridity index	*n*	median	mean	min	max	First quartile	Third quartile
0–0.5	1536	75.0	74.3	32.2	137.2	62.4	85.8
0.5–1	3559	61.8	62.0	3.8	136.7	50.9	72.5
1–1.5	3144	55.4	56.1	8.8	112.0	46.7	65.6
1.5–2	1392	52.5	54.3	19.7	108.4	44.6	63.6
2–5	1258	55.6	55.5	15.3	105.3	46.5	64.8

The number of observations (*n*) is shown as are distribution parameters (median, mean, minimum and maximum and lower and upper quartiles). AI < 1 indicates water-limited conditions. We excluded 32 observations for AI > 5.

In Methods, we provide a detailed analysis showing that *W* has a lower limit which corresponds to an upper limit on soil water potential (when soils are saturated). Using a combination of hydrological and biological theory, we show how this saturation point, and lower limit in *W*, relates to AI. Observations and theory both predict that stomatal conductance, and *W*, respond not to precipitation but rather to water availability (See Methods). Thus, *W* should decrease with rising precipitation (P) up to the point where the soil is water-saturated but should then be insensitive to further increases in P. Simple mass balance considerations predict that the threshold value of P (say P*) that saturates the soil corresponds approximately to an AI ≈ 1. Soil water content is determined by the balance among precipitation, runoff (*r*), drainage (*d*) and evapotranspiration (*T*). These terms are approximately in balance at annual time scales, so that P ≈ *T* + *r* + *d* and hence P/*T* ≈ 1 + (*r* + *d*)/*T*. When P is just sufficient to saturate the soil, *T* approximates PET, so that the value of AI (AI* = P*/PET) at this point is:

1 $${{{\tt{A}}}}{{{{\tt{I}}}}}^{\ast }=\frac{{\tt{P}}^{\ast }}{{{{\tt{PET}}}}}\approx {\tt 1}+\frac{\tt{r+d}}{{{{\tt{PET}}}}}$$

Thus, provided runoff and drainage are small relative to PET when P = P^*^, the threshold value of AI— above which *W* should be insensitive to AI—is ~1. Tree ring and climate observations (Fig. 2a–c) thus match with and link, fundamental hydrological and stomatal theory.

**Fig. 3:**
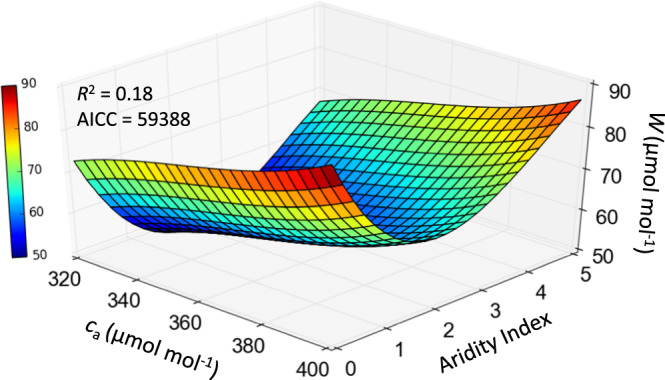
Effect of atmospheric CO_2_ (*c*_a_, µmol mol^−1^) and aridity index (precipitation/potential evapotranspiration, mm mm^−1^) on intrinsic water use efficiency (*W*, µmol mol^−1^) for the period 1965–2015. Polynomial regression of the combined effect of *c*_a_ and aridity index on *W*. Aridity index calculated using TerraClimate data. AICC denotes Akaike’s Information Criterion.

Three-way interactions among *c*_a_, AI and *W* are represented in Fig. 3. *W* was clearly greater in 2015 (*c*_a_ ≈ 395 ppm) than in 1965 (*c*_a_ ≈ 320 ppm; Fig. 3) for sites where AI < 1, and driven by increasing *c*_a_. Where 1 < AI < 4, changes in *W* with *c*_a_ were smaller and more variable—again in agreement with our theoretical analysis. There are relatively few sites with AI > 4 (Fig. 2) and the response surface in this range is uncertain owing to the shortage of data (Fig. 3).

### Interactive effects of CO_2_, climate and nitrogen deposition on *W*

The data also reveal changing relative influences of *c*_a_ and cumulative N deposition (20 years) on *W* over the 50-year study period (1965–2015). In the early decades (i.e. 1965–1985), the greatest recorded *W* were associated with fast rates of cumulative N deposition (Supplementary Fig. 4a). These clear effects of N deposition on *W* have faded since. During most recent decades (i.e. 1995–2015) positive effects of *c*_a_ on *W* were enhanced by low-moderate rates of N deposition (~20 gN m^−2^ 20 years^−1^; Supplementary Fig. 4a). The response surface varied little if annual N deposition or lagged annual deposition were used in place of cumulative N deposition (Supplementary Fig. 4b, c).

**Fig. 4:**
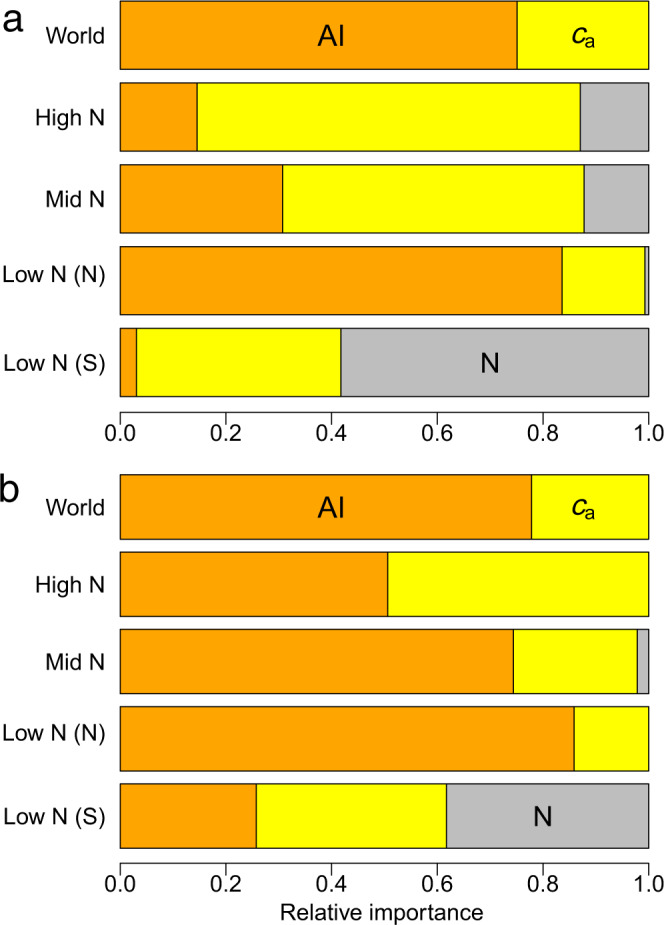
Proportional contributions to variance due to fixed effects in multivariate models (see Table 2) of *W* (µmol mol^−1^) based on atmospheric CO_2_ (*c*_a_, µmol mol^−1^), aridity index (mm mm^−1^), and cumulative nitrogen deposition (g N m^−2^ 20 years^−1^), for the period 1965–2015. Climatic parameters were derived from **a** TerraClimate database and **b** CRU database.

When included as fixed effects in best-fit, multivariate mixed models for *W* (Table 2), the explanatory value of AI and cumulative N deposition consistently improved from high N zone to low N zone (see Methods for definition of N deposition zones). The top ten considered models based on Terraclimate, and their corresponding AIC, are shown in Supplementary Table 1. Tree-to-tree variation in *W* within study sites (included in conditional *R*^2^) dominated explained variance. Coefficients of multivariate models were mostly highly significant (*p* < 0.001) for both climate databases (Table 2). The explanatory power (relative importance) of *c*_a_, AI and cumulative N deposition in multivariate models are shown in Fig. 4. Only in the low N (south) zone was cumulative N deposition a major contributor, irrespective of climate database. For all other N deposition zones, *c*_a_ and AI contributed a minimum of 85% of the marginal *R*^2^ (Fig. 4 and Table 2). Contributions of AI increased from the high N zone to the low N zone.

**Table 2:** Best-fit multivariate analysis for *W* (µmol mol^−1^) as a function of atmospheric [CO_2_] (*c*_a_, µmol mol^−1^), aridity index (AI = P/PET, dimensionless) and cumulative N deposition (N, gN m^−2^ 20 years^−1^) as fixed effects.

N zone	Database	Equation	*n*	Marginal *R*^2^	Conditional *R*^2^
World	TerraClimate	*W* = ac_a_ + b*c*_a_^2^ + cAI + dAI^2^ + eAI^3^ + I	10921 (411)	0.16	0.73
High N	TerraClimate	*W* = a*c*_a_ + bAI + cN + dN^2^ + I	4557 (166)	0.09	0.80
Mid N	TerraClimate	*W* = a*c*_a_ + bAI + cA^2^ + dN + I	2111 (75)	0.10	0.82
Low N (North)	TerraClimate	*W* = a*c*_a_ + bAI + cA^2^ + dN + I	3350 (131)	0.22	0.71
Low N (South)	TerraClimate	*W* = a*c*_a_ + b*c*_a_^2^ + cN + dN^2^ + I	903 (39)	0.24	0.64
World	CRU	*W* = a*c*_a_ + b*c*_a_^2^ + cAI + dAI^2^ + I	10921 (411)	0.21	0.70
High N	CRU	*W* = a*c*_a_ + b*c*_a_^2^ + cAI + dAI^2^ + I	4557 (166)	0.14	0.73
Mid N	CRU	*W* = a*c*_a_ + bAI + cN + I	2111 (75)	0.22	0.71
Low N (North)	CRU	*W* = a*c*_a_ + bAI + cAI^2^ + I	3350 (131)	0.26	0.67
Low N (South)	CRU	*W* = a*c*_a_^2^ + b*c*_a_^3^ + cAI + dN^3^ + I	903 (39)	0.28	0.59

N zone	Database	Coefficients					
		*a*	*b*	*c*	*d*	*e*	I
World	TerraClimate	0.844	−8.71E-04	−10.3	2.23	−0.136	−118
High N	TerraClimate	0.132	−3.67	1.43	−0.0257		1.09*
Mid N	TerraClimate	0.171	−6.24	0.512	0.455		1.15*
Low N (North)	TerraClimate	0.212	−8.54	1.50	0.711		−3.59*
Low N (South)	TerraClimate	0.261	−1.46*	2.03*	−0.178		−32.4
World	CRU	0.813	−8.23E-04	−8.87	1.28		−114
High N	CRU	1.553	−1.91E-03	−8.81	1.20		−241
Mid N	CRU	0.171	−4.36	0.458			−1.04*
Low N (North)	CRU	0.255	−12.1	2.43			−13.2
Low N (South)	CRU	1.88E-03	−2.80E-06*	−2.353*	−5.66E-03		−45.9*

I = intercept. Separate results are presented for the CRU and TerraClimate databases (see Methods). Unless noted otherwise, all models, intercepts and coefficients (a–e) were significant at *p* < 0.001 (using two-sided *t*-tests with no adjustments made). Shaded coefficients are significant at *p* < 0.01, and non-significant coefficients (*p* > 0.01) are marked with an asterisk. Northern hemisphere sites (N) span high, mid and low N deposition while southern hemisphere sites (S) are all low N. *n* = the total number of observatio*n*s of *W*, with the total number of trees in parentheses. Marginal *R*^2^ includes fixed effects only, while Conditional *R*^2^ includes variation between trees (random effects).

### Influences of other sources of variation

There were only small variations in models reported in Table 2 if we included a correction for effects of CO_2_ on PET (Supplementary Tables 2 and 3, see Methods). Likewise, if we replaced AI with its constituent drivers (P and PET) as independent sources of variation (Supplementary Table 4 and Supplementary Fig. 5), overall patterns were unchanged irrespective of climate database—cumulative N deposition was a minor contributor except in the low N zones while *c*_a_, P  and PET dominated explained variance. If we replaced AI by VPD, explained variance fell to less than 10% (Supplementary Table 5). There has been a relatively short period for which there have been consistent increases in VPD (the last 20–30 years in the last 100 years, See Supplementary Fig. 6).

## Discussion

The data show a clear threshold for the dependence of *W* on AI, with no apparent dependence in wetter ecosystems, above AI ≈ 1. As far as we are aware, this is the first time an ‘aridity threshold’ for climatic control of a major physiological process has been demonstrated empirically at a global scale. The threshold and our analysis Eq. (8) suggest the cost to plants of further stomatal opening is independent of precipitation when AI >> 1. By implication, stomatal conductance is likely far less constrained by evolved anatomical/morphological or physiological traits (e.g. density, maximum aperture and structure of stomata; duration of stomatal opening, the extent of opening) when AI > 1 than when AI < 1. Including consideration of autocorrelation and within-site variability (Fig. 2), we show that annual *W* increases from ~60 to <80 as AI declines from ~1 to 0.1. A significant curvilinear relationship (e.g. negative exponential) would also fit the entire aggregated data set, albeit this would obscure the threshold—a notorious consequence of data aggregation^21^. The relationship of *W* to AI—including the aridity threshold—can be heuristically modelled using an optimisation approach to stomatal behaviour and biomass partitioning (See Methods: Eq. (1); Eqs. (5)–(11). Our model also predicts that *W* and AI are negatively related where 0 < AI < 1 and unrelated where AI > 1. These predictions add a global perspective to more regional studies. For example, Givnish et al.^22^ demonstrated a linear relationship between *W* and a scalar for water availability similar to that used here (P/*E*_*p*_, where *E*_*p*_ = pan evaporation), over a much more limited geographic range in Australia. For some tropical forests that span a large range of AI, long-term rates of change in *W* (per unit time or per unit *c*_a_) were negatively related to *P*^23^.

The data and relationships uncovered here help reconcile and clarify reports of positive, but varying-strength effects of *c*_a_ on *W* for forests across climatic zones, as well as differences in effects of *c*_a_ on *W* amongst life forms (e.g. Gymnosperms vs Angiosperms, evergreen vs deciduous). For example, several studies have reported that water availability plays a major role in determining *W* at annual scales^24,25^. Annual *W* for forests dominated by broad-leaved evergreen and needleleaf trees is typically greater than for forests where broad-leaved deciduous trees are dominant^26^, while *W* has increased more rapidly with *c*_a_ in evergreen species than in deciduous species^27^. Leaf-level evidence suggests that gymnosperms show consistently less sensitivity of stomatal conductance to *c*_a_ than angiosperms^28^, although gymnosperms also show consistently greater *W* (see also Supplementary Fig. 3). Nonetheless, differences in geographic distributions of species based on phylogeny (Angiosperms vs Gymnosperms) or life-form (evergreen vs deciduous) can confound efforts to predict *W* on the same bases, given their interdependence^29,30^. The threshold for AI-driven variation in *W* revealed here helps distinguish climatic from phylogenetic or life-form influences.

The overwhelming majority of flux-based (e.g. ref. ^24^) and tree ring-based (e.g. ref. ^31^) analyses of *W* have been undertaken in central Europe and the eastern USA—two regions where both atmospheric N inputs are historically large (See Fig. 1) and AI > 1. Conclusions arising from these studies and regions that increased *W* is driven more by enhanced photosynthesis than by reduced stomatal conductance (e.g. ref. ^31^) are not surprising given the reliance of photosynthesis on leaf N. However, we show that once variation among trees at study sites is accounted for (Table 2 and Fig. 4), AI and *c*_a_ account for nearly all the remaining variance in annual *W* (residual variance typically ~10%) for the northern hemisphere, irrespective of N deposition zone. We urge caution in extending conclusions that enhanced photosynthesis is driving rises in *W* beyond central Europe and the eastern USA. The data assembled here suggest AI plays a far stronger role in determining *W* for most of the northern hemisphere (i.e. outside central Europe and the northeastern USA; Table 2 and Fig. 4). Forests classed as arid dominate most of the western half of the USA, much of Asia and most of the Southern Hemisphere outside the wet tropics. Guerrieri et al.^31^ noted that reductions in stomatal conductance with rising *c*_a_ are more apparent in species that experience moisture limitations—a point entirely consistent with an aridity threshold. Similarly, geographic restriction of data constrains conclusions with respect to life forms (e.g. ref. ^26^). An important implication of the present study is that many present hydroclimate models need adjusting to account for the aridity threshold, especially when applied at scales that incorporate large ranges in AI—for example, tropical river basins such as the Congo and Amazon. Responses of *W* to CO_2_ are now routinely included as drivers of rainfall and runoff in models designed to predict regional and continental-scale hydrological outcomes (refs. ^14,15,32,33^). Including a threshold for climate dependence of *W* should—at least theoretically—help improve the performance of hydroclimate models.

Increasingly well-known patterns of long-distance transport of atmospheric pollutants suggest large areas of Northern Hemisphere forests were subject to N pollution for much of the 20th century. Our tree ring-based analyses suggest that for the period 1965–2015, N deposition had modest effects on *W*. Effects were greatest in the period 1965–1980 (e.g. Supplementary Fig. 5) when *c*_a_ < 360 µmol mol^−1^ and have moderated since. This may be contrasted with increasingly compelling evidence that leaf nitrogen concentrations have mostly been falling in recent decades^8,9^. Using N isotope data in tree rings, MacLauchlan et al.^34^ added support for this view, at least for US forests, with trajectories of decline in N availability that were independent of N deposition. A reasonable summary is that rising CO_2_ and temperature are leading to reduced N availability, which is then reinforced by the success of recent global efforts to reduce N emissions and thus N deposition (see for example ref. ^35–38^). A cautionary note is that N deposition impacts are often greatest at relatively low rates for a range of ecosystem functions^39,40^, as seen in the low N Zones in the Southern Hemisphere (see Fig. 4 and Supplementary Fig. 5). Further caution is warranted since N deposition at greater rates can also affect tree growth indirectly (e.g. through soil acidification) with possible impacts on *W*. Nonetheless, policy and practical successes in reducing atmospheric N pollution—but not CO_2_ pollution or other greenhouse gases—ensure changes in aridity (and climate generally) are likely drivers of *g*_s_ and future changes in *W*, especially when coupled with slowing CO_2_-driven responses^4^.

There has been considerable recent attention given to rising VPD as a driver of plant water use^41,42^ and its interactions with other changes in climate. Based on the global data reported here, AI (a measure of overall water balance) was a better predictor of *W* than VPD over the long term. While VPD is an obvious driver of atmospheric water potential, soil water potential depends on soil depth and physical properties, both of which serve to buffer plant water use (see also ref. ^41^). Furthermore, a sustained increase in VPD has only been observed relatively recently (last 20–30 years, see Supplementary Fig. 6, also ref. ^42^). For the sites represented here, recent increases in VPD were uniformly distributed. However, rises in VPD have coincided with slowing rates of increase in *W*^4^ despite commentary^42^ that they should increase *W* (via induced stomatal closure). So far then, we have insufficient data to establish VPD as a globally significant driver of century scale (and perhaps shorter term) changes in *W*. Both small annual increases and decreases in *W* remain possible under conditions of rising *c*_a_, although large increases per unit *c*_a_ now seem less likely than they were during much of the last century^4^. Future changes in annual *W* will depend more on small relative changes in photosynthetic capacity (CO_2_- and light-saturated assimilation rate, *A*_max_) and *g*_s_. If leaf N (and *A*_max_) continue to fall, then rates at which *g*_s_ declines in response to rising *c*_a_ (possibly as a result of increases in VPD) will play key roles in determining *W* and its spatial and temporal variation.

In contrast to northern boreal/coniferous forests where the evidence supports an assumption of N limitation to physiology and growth^43^, there is much weaker evidence for other forests including those in the tropics and the Southern Hemisphere^44,45^ and phosphorus limitation is just as likely as nitrogen limitation. Much of the area of the Australian and African continents shows evidence of P-limitation in addition to water limitations, and for only relatively small regions on the west coasts of South America and Africa is there evidence of N limitation. One recent study^46^ has shown a decline in *W* associated with increases in N deposition to P-limited forest. Our analysis suggests this response could extend to other P-limited forests if *W* were measured at appropriate time scales. Increasing N inputs can presage phosphorus deficiency (or N:P imbalance), where declining *W*^47^ is due to reduced rates of photosynthetic carbon fixation^48,49^ rather than reduced stomatal conductance.

Not all variation in *W* is due to climate, *c*_a_ or N deposition. For Europe, Frank et al.^1^ noted that even in temperate climates, lengthened growing seasons, increases in leaf area and rising temperatures could all result in increased ecosystem-level transpiration, rather than less, and irrespective of gains in leaf-level *W* due in part to reduced stomatal opening. Similarly, temporary increases in carbon storage in semi-arid regions of the Southern Hemisphere were associated with expansions in the area of woody shrubs, rather than increases in *W* per se^50^. Single site studies (e.g. FACE) have been the primary underpinning of many models of carbon and water and are being augmented by an expanding network of flux stations to expand the populations of inference to larger portions of the planet. Yet most of the planet remains unrepresented by the flux networks. Tree ring data can provide fine-grained spatial coverage of *W*, as well as longitudinal measures of *W*, and help fill inevitable gaps in knowledge and data derived from other sources.

## Methods

### Calculation of *W*

We combined a recently compiled, global data set on intrinsic water use efficiency (*W*, based on the relative abundances of ^13^C and ^12^C in tree rings) with data on climate and atmospheric nitrogen inputs for global forests. The tree ring data are publicly available (10.1038/s41558-020-0747-7) and further details have been published (see ref. ^4^). In brief, the full data set contains 422 tree ring chronologies that collectively span from 1851–2015. Here we used a subset of 411 that span the period for which detailed climate and N deposition data are available. This corresponds to a 50-year period, 1965–2015.

The tree ring data set for *W* includes a traditional method of calculation of *W*, as originally developed by Farquhar et al.^51^:

2 $$W=\frac{A}{{g}_{s}}=\frac{{c}_{a}-{c}_{i}}{1.6}=\frac{{c}_{a}}{1.6}\left(1-\frac{{c}_{i}}{{c}_{a}}\right),$$

which can be re-written as:

3 $$\frac{{c}_{i}}{{c}_{a}}=\frac{\Delta -a}{b-a},$$

where *a* = 4.4‰ and *b* = 27‰. The ratio *c*_i_/*c*_a_ can be estimated from Δ, the δ^13^C of wood corrected for the isotopic composition of atmospheric CO_2_.

The data set also includes a second calculation, that follows Keeling et al.^52^, based on assumed influences on *W* of mesophyll conductance and photorespiration, and photosynthetic responses to *c*_a_:

4 $$\frac{{c}_{i}}{{c}_{a}}=\frac{\Delta -a+\left(b-{a}_{m}\right)\left(\frac{A}{{c}_{a}}\right)\frac{1}{{g}_{m}}+\frac{f{\Gamma }_{\ast }}{{c}_{a}}}{b-a}.$$

In Eq. (4), *a* ( = 4.4‰) and *b* ( = 30‰) are discrimination coefficients for stomatal diffusion and CO_2_ fixation by Rubisco, *a*_m_ = 1.8‰, *f* is the discrimination due to photorespiration (12‰), *g*_m_ is mesophyll conductance to CO_2_ (0.2 mol m^−2^ s^−1^) and Γ_*_ is the photorespiratory CO_2_ compensation point (43 µmol mol^−1^). A recent meta-analysis^53^ of effects of mesophyll conductance on isotope-derived *W* supports the Keeling et al.^52^ approach (not necessarily the scale of correction) adopted here.

### Climate, nitrogen deposition and CO_2_ data

#### Climate

As tree ring data on *W* are typically reported on an annual basis, we used annual average data for climate variables (see also main text). We used publicly available data on climate which are widely used and have been fully described^16,17^. CRU data has 0.5° spatial resolution and TerraClimate ~0.042°. While both datasets make similar use of primary data from weather stations, TerraClimate differs from CRU in consideration of topographic effects and use of remotely sensed climate input, and how these are represented in monthly climate surfaces (WorldClim 2, see ref. ^54^). We selected precipitation and PET owing to their direct role in AI. Both CRU and TerraClimate use a Penman–Monteith approach for calculating PET^16,17^. We also extracted data for VPD directly from TeraClimate. With CRU data, we calculated VPD using the August–Roche–Magnus approach^55^.

We tested if PET corrected for the effects of CO_2_ would affect our results. We used the formulation provided by Lian et al.^56^ who provide a detailed discussion of the known issues that accompany the use of Penman–Montieth approach to estimate PET (as used in both CRU and TerraClimate; see also Yang et al.^57^) and its use in aridity indices. Our study was restricted to trees and forests and woodlands, which removes some but not all of the variation in PET that results from the assumption of a consistent surface resistance (as found in grasslands).

#### Nitrogen deposition

We used N deposition data at 0.5° spatial resolution from ISIMIP (https://www.isimip.org; see^18,19^), which we then aggregated to annual time steps, summing wet and dry deposition, ammonium and nitrate. While not a measure of available N per se, atmospheric N deposition has been extensively studied in relation to tree and forest growth (e.g. ref. ^58^) and is equally extensively used in models to make predictions about future physiology and growth^59^. Geographically, nitrogen deposition from the atmosphere is strongly dependent on anthropogenic emissions of N to the atmosphere. Axiomatically, wet deposition depends on rainfall. Central Europe and the northeast USA are well-known for historically high rates of emissions and deposition. For large areas elsewhere—including continents such as Africa, South America and Australia— N emissions are comparatively low. Rapid recent increases in industrialisation—and use of combustion engines—in China, India and other Asian countries, have also increased emissions of N for that region^35^. Efforts to curb emissions have seen significant falls in N deposition in some areas. For example, current rates of N input to forest ecosystems in the northeastern USA are of the order of 0–10 kg ha^−1^ year^−1^ for both oxidised and reduced forms of N^36^ but NO_x_ inputs are now far less than they were 20 years ago, as a result of the Clean Air Act. There are similar spatial and temporal patterns in Europe^37^. In China especially^35^ (and in other parts of Asia^60^), rates of atmospheric N deposition rose sharply since the 1980s but then moderated in recent years via government policy^38^.

In consequence, rates of atmospheric N deposition are highly spatially and temporally heterogenous. Definition of so-called ‘critical loads’ thus vary according to ecosystem and author. As an example, and after a major data synthesis and analysis, Pardo et al.^61^ suggested that critical loads for USA ecoregions might vary between 1 and 39 kg N ha^−1^ year^−1^. Equally, there is ample evidence that cumulative N deposition is more ecologically significant than one-off additions.

Given the lack of consensus as to N deposition thresholds, and the known significance of cumulative N inputs in place of annual, we instead sought to geographically define nominal ‘N deposition zones’ that reflected the different histories of N deposition across the globe, as well as rates of deposition:

- High N; central Europe (45–60 N; 15 W–45 E) + eastern USA (23.5–50 N; 100–60 W)
- Medium N; mainly eastern Asia (0–45 N; 70–150 E)
- Low N; divided into
  - Northern Hemisphere (mostly dominated by coniferous forest in western North America (>100 W) and the boreal zone generally (>50 N in North America, >60 N elsewhere)
  - Southern Hemisphere (angiosperms are the most dominant tree forms).

To characterise these zones, we assessed N deposition at the site level (see Supplementary Figs. 7 and 8). Study sites in our High N zone averaged ~17 gN m^−2^ 20 years^−1^ (~8.5 kg N ha^−1^ year^−1^), those in Mid N zone averaged ~8 gN m^−2^ 20 years^−1^, while low N zones (northern or southern) averaged 3–5 gN m^−2^ 20 years^−1^ (Supplementary Fig. 7). N deposition at sites included in the high N and low N (south) zones were evenly (and normally) distributed. Annual N deposition for sites in the high N zone reflected an almost uniform history of steady increases from 1950 onwards, with a slowing increase from around 2000 (Supplementary Fig. 8). Study sites in the nominal Mid N zone showed a stronger rate of increase in annual N deposition from ~1980, with a little slowing in the rate of increase after 2000 (Supplementary Fig. 8). There is also a much wider range of annual N deposition for this nominal zone. Most sites in the low N zones (north or south) show slow increases in annual N deposition from 1950. However, in the northern low N zone, there is a much wider range of annual N deposition, including some sites with strong increases (e.g. China). A result of these temporal changes in annual N deposition at study sites classed as mid N or low N (north) is that average N deposition (over 20 years) for a considerable number of individual sites can be several-fold greater than the average for the whole deposition zone (a positive tail in data distribution; Supplementary Fig 7). While our nominal N deposition zones do not constitute a formal classification, we argue they represent a reasonable approach to a vexed issue, and more importantly that anomalies in data distribution have no effect on our statistical analysis or interpretation.

#### CO_2_

CO_2_ data were sourced as described by Adams et al.^4^. In brief, we used the CO_2_ data reported by Mc Caroll and Loader^62^, supplemented with additional, more recent data from Keeling et al.^63^. See Adams et al.^4^ for discussion of possible sources of variation in CO_2_.

### Heuristic model of the relationship between W and AI

Long-term shifts in stomatal conductance to CO_2_ (*g*_s_) and net CO_2_ assimilation rate (*A*) are determined by the balance between costs and benefits of biomass partitioning. The balance can be understood in economic terms as profit maximisation driven by natural selection. Carbon is invested to create and sustain a given hydraulic conductance (*K*), which enables water uptake and transport, and that water is then exchanged for CO_2_ through stomata. The profit of this carbon investment cycle is maximised if the marginal carbon revenue of water (∂*A*/∂*E*, where *E* is transpiration rate) equals the marginal carbon cost of acquiring that water (∂*C*/∂*E*, where *C* is the average annual carbon cost of building and maintaining the hydraulic infrastructure represented by *K*).

The marginal revenue of water, ∂*A*/∂*E*, is approximately:

5 $$\frac{\partial A}{\partial E}{{\,\approx\, }}\frac{A}{E}\left(\frac{k}{k+{g}_{s}}\right),$$

(see ref. ^64^) where *k* is the slope of the response of *A* to intercellular CO_2_ concentration (*c*_i_) when biochemical parameters of photosynthesis are held constant. A simple model for that response is *A* = *k*(*c*_i_ – Γ), where Γ is the CO_2_ compensation point. Similarly, the dependence of *A* on *g* is given by *A* = *g*_s_(*c*_a_ − *c*_i_), where *c*_a_ is ambient CO_2_ concentration. Combining these gives *k*/(*k* + *g*_s_) = *A*/(*g*(*c*_a_ − Γ)) = *W*/(*c*_a_ − Γ). Note also that *E* ≈ 1.6*g*_s_*D*, where *D* is the leaf-to-air water vapour mole fraction gradient, so that *A*/*E* = *A*/(1.6*Dg*_s_) = *W*/1.6*D*.

Substituting these expressions for *k*/(*k* + *g*_s_) and *A*/*E* into Eq. (5) gives:

6 $$\frac{\partial A}{\partial E}=\frac{{W}^{2}}{1.6D\left({c}_{a}-\Gamma \right)}.$$

The marginal carbon cost of water, ∂*C*/∂*E*, is

7 $$\frac{\partial C}{\partial E}={\left.\frac{\partial C}{\partial E}\right|}_{\psi {{{leaf}}}}+{\left.\frac{\partial C}{\partial \left(-{\psi }_{{{{leaf}}}}\right)}\right|}_{K}\frac{d\left(-{\psi }_{{{{leaf}}}}\right)}{{dE}}.$$

The first term on the right-hand side of Eq. (7) (∂*C*/∂*E* | *ψ*_leaf_) is the marginal carbon cost of increasing *E* by increasing whole-plant hydraulic conductance (*K*) at a given leaf water potential. Since *E* = *K*(*ψ*_soil_ − *ψ*_leaf_), where *ψ*_soil_ and *ψ*_leaf_ are soil and leaf water potentials, respectively, that marginal cost is 1/[(∂*E*/∂*K*)(∂*K*/∂*C*)] = 1/[(*ψ*_soil_ − *ψ*_leaf_)(∂*K*/∂*C*)]. The second term on the right-hand side of Eq. (7) represents the direct carbon costs resulting from any decrease in leaf water potential at a given hydraulic conductance. These costs may include, for instance, the cost of refilling embolized xylem conduits, as well as more abstract costs such as the risk of foregone photosynthesis due to permanent loss of hydraulic conductivity to embolism. We shall not specify these costs in any detail, except to note that they are expected to increase in magnitude as *ψ*_leaf_ becomes more negative. To facilitate interpretation of this term below, we will replace ∂*C*/∂(−*ψ*_leaf_) with a generic positive function of minus *ψ*_leaf_, thus: *f*^*+*^(−*ψ*_leaf_); this clarifies that the costs increase as *ψ*_leaf_ becomes more negative, and vice versa. Finally, note that *d*(−*ψ*_leaf_)/*dE* = 1/*K*, since *E* = *K*(*ψ*_soil_ − *ψ*_leaf_); this ignores effects of *ψ*_leaf_ on *K* itself, but such effects are generally small except when water stress is so large as to cause nearly complete stomatal closure^65^.

8 $$\frac{\partial C}{\partial E}=\frac{1}{\left({\psi }_{{{{soil}}}}-{\psi }_{{{{leaf}}}}\right)\frac{\partial K}{\partial C}}+\frac{{f}^{+}\left(-{\psi }_{{{{leaf}}}}\right)}{K}.$$

To identify the optimum, we set equal the marginal revenue and cost to give:

9 $$W=\sqrt{1.6D\left({c}_{a}-\Gamma \right)}\sqrt{\frac{1}{\left({\psi }_{{{{soil}}}}-{\psi }_{{{{leaf}}}}\right)\frac{\partial K}{\partial C}}+\frac{{f}^{+}\left(-{\psi }_{{{{leaf}}}}\right)}{K}}.$$

The analysis in the main text concerns the relation of *W* to AI, which directly reflects changes in *ψ*_soil_ (via precipitation and hence soil moisture). As we state there, stomatal conductance, and *W*, respond not to precipitation but rather to water availability (see also refs. ^66–69^).

To interpret the predictions of Eq. (9) in that context, we must first consider how *ψ*_leaf_ varies in relation to *ψ*_soil_. Plant species vary in the degree to which they allow leaf water potential to decline when soil water potential declines. This pattern can be described phenomenologically by a continuum between two extremes. At one end are species that are isohydrodynamic (*sensu*^70^) where *ψ*_leaf_ tracks *ψ*_soil_ such that the gradient (*ψ*_soil_ − *ψ*_leaf_) is independent of *ψ*_soil_, while at the other end of the continuum, isohydric species exhibit constant *ψ*_leaf_ with the gradient increasing in magnitude as *ψ*_soil_ increases.

Equation (9) predicts different responses of *W* to changes in soil water potential for these two species types. For isohydric species, the gradient (*ψ*_soil_ − *ψ*_leaf_) increases as *ψ*_soil_ increases, while *ψ*_λeaf_ and hence *f*^*+*^(−*ψ*_leaf_) remains constant, so that:

10 $$W\propto \sqrt{\frac{1}{{\psi }_{{{{soil}}}}+a}+b}$$

where *a* and *b* are positive factors that are not explicitly functions of *ψ*_soil_ but are expressed in this manner to highlight the predicted qualitative response of *W* to *ψ*_soil_. Equation (10) predicts that *W* decreases as *ψ*_soil_ increases for isohydric species.

For isohydrodynamic species, the gradient is constant as *ψ*_soil_ increases, but *f*^*+*^(−*ψ*_leaf_) decreases because *ψ*_leaf_ becomes less negative. It follows that

11 $$W\propto \sqrt{{a}^{{{{\prime} }}}-{f}^{+}\left({\psi }_{{{{soil}}}}-{b}^{{{{\prime} }}}\right)}$$

where, as in Eq. (10), *a*′ and *b*′ are positive factors that are not explicitly functions of *ψ*_soil_. Again, the model predicts that *W* decreases as *ψ*_soil_ increases for isohydrodynamic species, as for isohydric species.

Consider now the behaviour of *ψ*_soil_ in relation to precipitation and hence the AI. *ψ*_soil_ has an upper limit of zero when the soil is saturated. Hence *W* has a lower limit with respect to *ψ*_soil_. We can use a simple hydrological model to explore heuristically how this saturation point, and lower limit in *W*, should relate to the AI. As a result of simple mass balance, soil water content is determined by the balance among precipitation (P), runoff (*r*), drainage (*d*), and evapotranspiration (*T*). These terms are approximately in balance at annual time scales, so that P ≈ *T* + *r* + *d* and hence P/*T* ≈ 1 + (*r* + *d*)/*T*. When P is just sufficient to saturate the soil (say P = P^*^), *T* approximates PET, so that:

12 $$ \normalsize \frac{{\tt{P}}^{\ast }}{{{{\tt{PET}}}}}\approx {\tt{1}}+\frac{\tt{d+r}}{{{{\tt{PET}}}}}$$

The quantity at left is the value of the AI (AI = P/PET) at which the soil reaches saturation so that *ψ*_soil_ stops increasing with AI, and *W* stops declining with AI. The quantity at right is approximately unity, provided that drainage and runoff are small relative to PET when P = P^*^.

In summary, our heuristic analysis suggests that *W* should decline until AI reaches a value close to unity and that *W* should then remain approximately constant as AI increases above that threshold.

### Statistical analysis

We used multivariate analyses to test the impact of climate variables and atmospheric N deposition on *W*— *c*_a_ relationships (CurveExpert 2.6.5) using Akaike’s Information Criterion (AICC) to determine the best model fit. We used R software (version 4.0.3) for further computations and visualisation and R libraries glmulti, segmented and lme4 for breakpoint analysis, segmented regression (Fig. 2) and fitting mixed models respectively (see also refs. ^71–74^). Further, we used mixed multivariate models (testing all possible combination of variables, no stepwise regression) to apportion variation in *W* to contributions of climate variables, atmospheric N deposition and atmospheric CO_2_. PET, P, VPD, cumulative N deposition (20 years), AI and atmospheric CO_2_ were the variables selected. Tree ring data for *W* were matched to CO_2_ N deposition and climate covariates. We tested lagged covariates, which did not improve our model results. We computed marginal and conditional coefficients of determination. We tested site (study site location) and tree (identified by species, some sites had several coexisting species) and then used a tree as a random effect in mixed models. We first used the CRU climate dataset (0.5° spatial resolution). We subsequently used the TerraClimate dataset (0.042° spatial resolution). We also repeated our assessment of best-fit models (selected using AICC) using TerraClimate after (a) replacing PET and P with AI, (AI = P/PET) and (b) replacing PET and P with VPD. We used in our analysis only the model with the lowest AIC (if several models had the same AIC, we used the model with the least covariates) and provide in the Supplement details of the ten best models. We calculated *P* values for the mixed models, approximated using a normal distribution. We limited our models to the most important variables considering up to third order polynomial covariates and derived relative importance using ANOVA. We calculated models for the whole dataset (*n* = 10,921) and separately for the four N deposition regions (in total 20 combinations, five regions × two climate datasets × 2 AI vs. P and PET). In addition, we calculated five combinations using VPD, and a further five combinations using AI corrected for changes in CO_2_ concentrations (*sensu*^56^). We estimated air pressure based on elevation and average temperature as an average of the maximum and minimum temperatures, to collate the input required for applying the Penman–Monteith model.

### Reporting summary

Further information on research design is available in the Nature Research Reporting Summary linked to this article.

## Supplementary information

Supplementary Information File

Reporting Summary

## Data Availability

We used a total of four publicly available data sets. Our recently compiled tree ring data is available at: 10.5281/zenodo.3693240. For climate, the Terraclimate data are available at: 10.1038/sdata.2017.191 while the CRU data are available at: 10.1038/s41597.020.0453.3. Finally, the nitrogen deposition data were extracted from the ISIMIP project as described in detail in Methods (see: https://www.isimip.org).

## Code availability

We used R software for computations and visualisation. R library glmulti (see https://CRAN.R-project.org/package=glmulti) was used for model selection and library lme4 (see https://github.com/lme4/lme4/) was used for fitting mixed models.
