# Supplementary material for: CO2, nitrogen deposition and a discontinuous climate response drive water use efficiency in global forests
Source: Nat Commun. 2021 Aug 31;12:5194. doi: 10.1038/s41467-021-25365-1 (PMC8408268; doi:10.1038/s41467-021-25365-1)
Supplement: Supplementary file 1 — Supplementary Information File [file 41467_2021_25365_MOESM1_ESM.pdf]

Supplementary Information: CO_2, nitrogen deposition and a discontinuous climate response drive water use efficiency in global forests.

Supplementary Tables 1 - 5
Supplementary Fig. 1 to Supplementary Fig. 8

Supplementary Table 1. Rank and AIC for 10 best models for *W* using as fixed effects: atmospheric [CO_2] (c_a, μmol mol^{-1}), Aridity Index (AI = P/PET, dimensionless) from Terraclimate database, and cumulative N deposition (N, gN m^{-2} 20 years^{-1}). The best model (Rank 1) corresponds to the model shown in Table 2.

Region	Rank	Model	AIC	Delta AIC
Global	1	$W = ac_a + bc_a^2 + cAI + dAI^2 + eAI^3 + I$	69422.6	0.0
	2	$W = ac_a + bc_a^2 + cAI + dN + eN^2 + I$	69425.5	2.9
	3	$W = ac_a + bc_a^3 + cAI + dN + eN^2 + I$	69425.5	2.9
	4	$W = ac_a + bc_a^2 + cc_a^3 + dAI + eN + fN^2 + I$	69425.5	2.9
	5	$W = ac_a + bAI + cN + dN^2 + I$	69425.5	2.9
	6	$W = ac_a + bAI + cAI^2 + dAI^3 + I$	69435.0	12.4
	7	$W = ac_a + bc_a^3 + cAI + dAI^2 + eAI^3 + I$	69436.9	14.2
	8	$W = ac_a + bc_a^2 + cc_a^3 + dAI + eAI^2 + fAI^3 + I$	69443.9	21.3
	9	$W = ac_a^2 + bAI + cN + dN^2 + I$	69447.4	24.8
	10	$W = ac_a^2 + bc_a^3 + cAI + dN + eN^2 + I$	69447.4	24.8
High N	1	$W = ac_a + bAI + cN + dN^2 + I$	28790.0	0.0
	2	$W = ac_a + bc_a^2 + cc_a^3 + dAI + eN + fN^2 + I$	28790.0	0.0
	3	$W = ac_a + bAI + cN + dN^2 + I$	28790.0	0.0
	4	$W = ac_a + bc_a^3 + cAI + dN + eN^2 + I$	28790.0	0.0
	5	$W = ac_a^2 + bc_a^3 + cAI + dN + eN^2 + I$	28807.4	17.3
	6	$W = ac_a^2 + bAI + cN + dN^2 + I$	28807.4	17.3
	7	$W = ac_a + bc_a^2 + dAI + eAI^2 + fN^3 + I$	28808.7	18.6
	8	$W = ac_a + bc_a^2 + cc_a^3 + dAI + eAI^2 + fN^3 + I$	28808.7	18.6
	9	$W = ac_a + bAI + cN + dN^3 + I$	28809.1	19.1
	10	$W = ac_a + bc_a^2 + cc_a^3 + dAI + eN + fN^3 + I$	28809.1	19.1
Middle N	1	$W = ac_a + bAI + cAI^2 + dN + I$	13008.7	0.0
	2	$W = ac_a^2 + bAI + cAI^2 + dN + I$	13021.2	12.5
	3	$W = ac_a + bAI + cAI^3 + dN + I$	13021.2	12.5
	4	$W = ac_a + bc_a^2 + cAI + dAI^2 + eN + I$	13024.3	15.6
	5	$W = ac_a + bAI + cAI^2 + dN^2 + I$	13026.2	17.5
	6	$W = ac_a^2 + bAI + cAI^3 + dN + I$	13033.7	25.0
	7	$W = ac_a^3 + bAI + cAI^2 + dN + I$	13034.2	25.5
	8	$W = ac_a + bc_a^2 + cAI + dAI^3 + eN + I$	13036.7	28.0
	9	$W = ac_a + bc_a^3 + cAI + dAI^2 + eN + I$	13038.2	29.5
	10	$W = ac_a + bAI + cAI^3 + dN^2 + I$	13038.6	29.9
Low N North	1	$W = ac_a + bAI + cA^2 + dN + I$	21447.7	0.0
	2	$W = ac_a + bAI + cN + dN^2 + I$	21460.0	12.3
	3	$W = ac_a + bc_a^3 + cAI + dN + eN^2 + I$	21460.0	12.3
	4	$W = ac_a + bc_a^2 + cAI + dN + eN^2 + I$	21460.0	12.3
	5	$W = ac_a + bc_a^3 + cAI + dN + eN^3 + I$	21460.2	12.5
	6	$W = ac_a + bc_a^2 + cAI + dN + eN^3 + I$	21460.2	12.5
	7	$W = ac_a + bAI + cN + dN^3 + I$	21460.2	12.5
	8	$W = ac_a + bAI + cAI^2 + dAI^3 + I$	21460.4	12.7
	9	$W = ac_a^2 + bAI + cAI^2 + dN + I$	21461.5	13.8
	10	$W = ac_a + bc_a^2 + cAI + dAI^2 + eN + I$	21464.0	16.3
Low N South	1	$W = ac_a + bc_a^2 + cN + dN^2 + I$	5895.4	0.0
	2	$W = ac_a + bAI + cN + dN^2 + I$	5895.4	0.0
	3	$W = ac_a + bc_a^3 + cAI + dN + eN^2 + I$	5895.4	0.0
	4	$W = ac_a + bAI + cAI^2 + dN^2 + I$	5897.7	2.3
	5	$W = ac_a + bN + cN^2 + I$	5898.4	3.0
	6	$W = ac_a + bAI + cAI^2 + dAI^3 + I$	5899.2	3.8
	7	$W = ac_a + bAI + cAI^2 + dN^3 + I$	5899.8	4.4
	8	$W = ac_a + bAI + cN^2 + I$	5900.4	5.0
	9	$W = ac_a + bAI + cAI^2 + dN + I$	5900.6	5.2
	10	$W = ac_a + bc_a^2 + cAI + dN + eN^3 + I$	5901.8	6.4

Supplementary Table 2. Best-fit mixed model analysis for *W* (µmol mol^{-1}) using as fixed effects: atmospheric [CO_2] (c_a, µmol mol^{-1}), Aridity Index (AI = P/PET, dimensionless; PET adjusted for effects of CO_2, see Methods), and cumulative N deposition (N, g N m^{-2} 20 $years^{-1}$).   I = intercept. Results are shown for TerraClimate database. Unless noted, all models, intercepts and coefficients (a-d) were significant at p<0.001 (using two-sided t-tests with no adjustments made). Shaded coefficients are significant at p<0.01, and non-significant coefficients (p>0.01) are marked with an asterisk. Northern hemisphere sites (N) span High, Mid and Low N deposition while southern hemisphere sites (S) are all Low N. *n* = the total number of observations of *W*, with the total number of trees in parentheses. Marginal R^2 includes fixed effects only (see Equation), while Conditional R^2 includes variation between trees (random effects).

N zone	*Equation*	*n*	*Marginal* R^2	*Conditional* R^2
World	$W = ac_a + bAI + cAI^2 + dAI^3 + I$	10921 (411)	0.17	0.73
High N	$W = ac_a + bc_a^2 + cAI + dN^3 + I$	4557 (166)	0.09	0.78
Mid N	$W = ac_a + bAI + cAI^2 + dN + I$	2111 (75)	0.16	0.77
Low N (North)	$W = ac_a + bAI + cAI^2 + dN + I$	3350 (131)	0.22	0.71
Low N (South)	$W = ac_a + bAI + cN^2 + I$	903 (39)	0.25	0.63

N zone	*Coefficients*				
	a	*b*	*c*	*d*	I
World	0.237	-10.11	2.15	-0.13	-12.43
High N	1.98	-0.0025	-3.73	-0.0002	-325.7
Mid N	0.172	-6.21	0.51	0.454	0.73
Low N (North)	0.211	-8.44	1.39	0.700	-3.61
Low N (South)	0.305	0.017	-0.08		-63.31

Supplementary Table 3. Rank and AIC for 10 best models for *W* using as fixed effects: atmospheric [CO_2] (c_a, μmol mol^{-1}), Aridity Index (AI = P/PET, dimensionless; PET adjusted for effects of CO_2, see Methods), and cumulative N deposition (N, gN m^{-2} 20 years^{-1}). The best model (Rank 1) corresponds to the model shown in Supplementary Table 2.

Region	Rank	Model	AIC	Delta AIC
Global	1	$W = ac_a + bAI + cAI^2 + dAI^3 + I$	69434.3	0.0
	2	$W = ac_a + bc_a^3 + cAI + dAI^2 + eAI^3 + I$	69434.3	0.0
	3	$W = ac_a + bc_a^2 + cAI + dAI^2 + eAI^3 + I$	69434.3	0.0
	4	$W = ac_a + bc_a^2 + cAI + dAI^2 + eN + I$	69454.8	20.5
	5	$W = ac_a + bAI + cAI^2 + dN + I$	69454.8	20.5
	6	$W = ac_a + bc_a^3 + cAI + dAI^2 + eN + I$	69454.8	20.5
	7	$W = ac_a + bc_a^2 + cAI + dAI^2 + I$	69463.8	29.5
	8	$W = ac_a^2 + bc_a^3 + cAI + dAI^2 + eAI^3 + I$	69464.3	30.0
	9	$W = ac_a^2 + bAI + cAI^2 + d\,AI^3 + I$	69475.2	40.9
	10	$W = ac_a + bAI + cAI^2 + I$	69475.2	40.9
High N	1	$W = ac_a + bc_a^2 + cAI + dN^3 + I$	28838.7	0.0
	2	$W = ac_a + bc_a^2 + cc_a^3 + dAI + eN^3 + I$	28838.7	0.0
	3	$W = ac_a + bc_a^2 + cAI + dAI^2 + I$	28841.7	3.0
	4	$W = ac_a + bc_a^3 + cAI + dN^3 + I$	28846.9	8.2
	5	$W = ac_a + bc_a^2 + cAI + dN^2 + I$	28851.2	12.5
	6	$W = ac_a + bc_a^2 + cc_a^3 + dAI + eN^2 + I$	28853.0	14.3
	7	$W = ac_a + bc_a^3 + cAI + dAI^2 + I$	28853.6	14.9
	8	$W = ac_a + bc_a^2 + cAI + dAI^3 + I$	28853.6	14.9
	9	$W = ac_a + bc_a^2 + cc_a^3 + dAI + eAI^2 + I$	28855.9	17.2
	10	$W = ac_a^2 + bc_a^3 + cAI + dN^3 + I$	28856.6	17.9
Middle N	1	$W = ac_a + bAI + cAI^2 + dN + I$	13008.2	0.0
	2	$W = ac_a + bc_a^3 + cAI + dAI^2 + eN + I$	13008.2	0.0
	3	$W = ac_a + bc_a^2 + cAI + dAI^2 + eN + I$	13008.2	0.0
	4	$W = ac_a^2 + bAI + cAI^2 + d\,N + I$	13020.7	12.5
	5	$W = ac_a^2 + bc_a^3 + cAI + dAI^2 + eN + I$	13020.7	12.5
	6	$W = ac_a + bc_a^3 + cAI + dAI^3 + eN + I$	13020.7	12.6
	7	$W = ac_a + bAI + cAI^3 + d\,N + I$	13020.7	12.6
	8	$W = ac_a + bc_a^2 + cAI + dAI^3 + eN + I$	13020.7	12.6
	9	$W = ac_a^2 + bAI + cAI^3 + d\,N + I$	13031.5	23.3
	10	$W = ac_a^2 + bc_a^3 + cAI + dAI^3 + eN + I$	13031.5	23.3
Low N North	1	$W = ac_a + bAI + cAI^2 + d\,N + I$	21447.2	0.0
	2	$W = ac_a + bc_a^2 + cAI + dAI^2 + eN + I$	21447.2	0.0
	3	$W = ac_a + bc_a^3 + cAI + dAI^2 + eN + I$	21447.2	0.0
	4	$W = ac_a + bc_a^2 + cAI + dAI^2 + eAI^3 + I$	21459.9	12.7
	5	$W = ac_a + bAI + cAI^2 + d\,AI^3 + I$	21459.9	12.7
	6	$W = ac_a + bc_a^3 + cAI + dAI^2 + eAI^3 + I$	21459.9	12.7
	7	$W = ac_a^2 + bAI + cAI^2 + d\,N + I$	21461.0	13.8
	8	$W = ac_a^2 + bc_a^3 + cAI + dAI^2 + eN + I$	21461.0	13.8
	9	$W = ac_a + bc_a^3 + cAI + dAI^3 + eN + I$	21472.6	25.4
	10	$W = ac_a + bc_a^2 + cAI + dAI^3 + eN + I$	21472.6	25.4
Low N South	1	$W = ac_a + bN + cN^2 + I$	5898.4	0.0
	2	$W = ac_a + bAI + cAI^2 + d\,AI^3 + I$	5899.7	1.3
	3	$W = ac_a + bc_a^2 + cAI + dAI^2 + eAI^3 + I$	5899.7	1.3
	4	$W = ac_a + bc_a^3 + cAI + dAI^2 + eAI^3 + I$	5899.7	1.3
	5	$W = ac_a + bAI + cN^2 + I$	5900.3	2.0
	6	$W = ac_a + bc_a^3 + cAI + dAI^2 + eN + I$	5900.7	2.4
	7	$W = ac_a + bc_a^2 + cAI + dAI^2 + eN + I$	5900.7	2.4
	8	$W = ac_a + bAI + cAI^2 + dN + I$	5900.7	2.4
	9	$W = ac_a + bAI + cN + I$	5902.5	4.1
	10	$W = ac_a + bAI + cN^3 + I$	5902.8	4.4

Supplementary Table 4. Best-fit mixed model analysis for *W* (µmol mol^{-1}) using as fixed effects: atmospheric [CO_2] (c_a, µmol mol^{-1}), potential evapotranspiration (PET, mm year^{-1}), precipitation (P, mm year^{-1}), and cumulative nitrogen deposition (N, g N m^{-2} 20 years^{-1}). I = intercept. Separate results are presented for the CRU and TerraClimate databases (see Methods). For further details see SI Table 2.

N zone	Database	*Equation*	*n*	*Marginal* R^2	*Conditional* R^2
World	*TerraClimate*	$W = ac_a + bPET + cP + dP^2 + I$	10921 (411)	0.14	0.77
High N	*TerraClimate*	$W = ac_a + bPET + cN + dN^2 + I$	4557 (166)	0.08	0.80
Mid N	*TerraClimate*	$W = ac_a + bPET + cPET^2 + dN + I$	2111 (75)	0.08	0.85
Low N (North)	*TerraClimate*	$W = ac_a + bPET + cN + dN^2 + I$	3350 (131)	0.16	0.78
Low N (South)	*TerraClimate*	$W = ac_a + bPET + cN + dN^2 + I$	903 (39)	0.25	0.64
World	*CRU*	$W = ac_a + bPET + cN + dN^2 + I$	10921 (411)	0.11	0.81
High N	*CRU*	$W = ac_a + bPET + cN + dN^2 + I$	4557 (166)	0.12	0.76
Mid N	*CRU*	$W = ac_a + bPET + cPET^2 + dN + I$	2111 (75)	0.22	0.72
Low N (North)	*CRU*	$W = ac_a + bPET + cN+ dN^2 + I$	3350 (131)	0.19	0.74
Low N (South)	*CRU*	$W = ac_a + bN + cN^2 + I$	903 (39)	0.21	0.67

N zone	Database	*Coefficients*				
		a	*b*	*c*	*d*	I
World	*TerraClimate*	0.232	0.013	-0.010	1.93E-06	-23.5
High N	*TerraClimate*	0.102	0.021	1.55	-0.027	-11.1
Mid N	*TerraClimate*	0.155	0.057	-2.57E-05	0.473	-25.2
Low N (North)	*TerraClimate*	0.183	0.015	1.86	-0.049	-16.6
Low N (South)	*TerraClimate*	0.274	0.016	1.69 *	-0.170	-56.4
World	*CRU*	0.184	0.021	0.956	-0.020	-27.9
High N	*CRU*	0.087	0.025	1.55	-0.026	-9.8
Mid N	*CRU*	0.156	0.094	-4.24E-05	0.467	-45.5
Low N (North)	*CRU*	0.188	0.021	1.71	-0.044	-24.5
Low N (South)	*CRU*	0.263	2.120*	-0.184		-35.2

Supplementary Table 5. Best-fit mixed-model analysis for *W* (µmol mol^{-1}) using as fixed effects: atmospheric [CO_2] (c_a, µmol mol^{-1}), vapour pressure deficit (VPD, kPa) and cumulative N deposition (N, g N m^{-2} 20 $years^{-1}$). I = intercept. Climate data are based on TerraClimate database (see Methods). For further details see SI Table 2.

N zone	Database	*Equation*	n	*Marginal* R^2	*Conditional* R^2
World	*TerraClimate*	$W = ac_a + bVPD + cN + dN^2 + I$	10921 (411)	0.07	0.84
High N	*TerraClimate*	$W = ac_a + bVPD + cN + dN^2 + I$	4557 (166)	0.08	0.80
Mid N	*TerraClimate*	$W = ac_a + bVPD + cVPD^2 + dN + I$	2111 (75)	0.11	0.82
Low N (North)	*TerraClimate*	$W = ac_a + bVPD + cN + dN^3 + I$	3350 (131)	0.06	0.86
Low N (South)	*TerraClimate*	$W = ac_a + bc_a^3 + cVPD^2 + dN^3 + I$	903 (39)	0.22	0.65

N zone	Database	*Coefficients*				
		a	*b*	*c*	*d*	I
World	*TerraClimate*	0.188	15.69	0.978	-0.021	-18.83
High N	*TerraClimate*	0.101	19.3	1.53	-0.027	-2.46*
Mid N	*TerraClimate*	0.168	35.1	-23.6	0.436	-14.2
Low N (North)	*TerraClimate*	0.188	16.66	1.47	-1.55E-03	-14.8
Low N (South)	*TerraClimate*	5.89E-01	-8.20E-07	5.51	-5.66E-03	-113

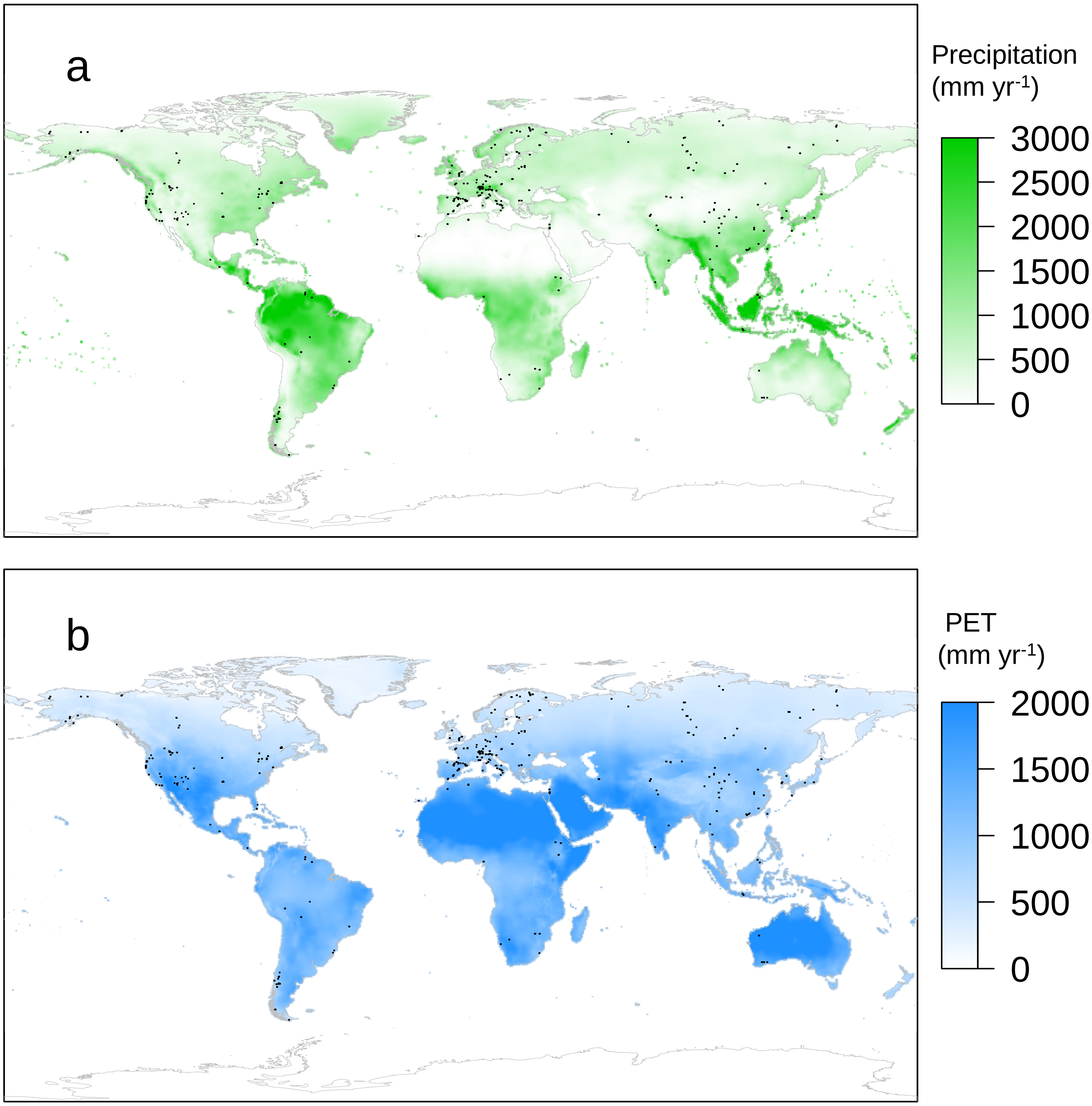

Supplementary Fig. 1 Geographic locations for 349 study sites in relation to mean precipitation (*P*) and potential evapotranspiration (PET). (a) Precipitation (mm year^{-1}). (b) Potential evapotranspiration (mm year^{-1}). Climate data were extracted from either TerraClimate or CRU databases (see Methods). For visibility, *P* was truncated at 3000 mm year^{-1} and PET at 2000 mm year^{-1}.

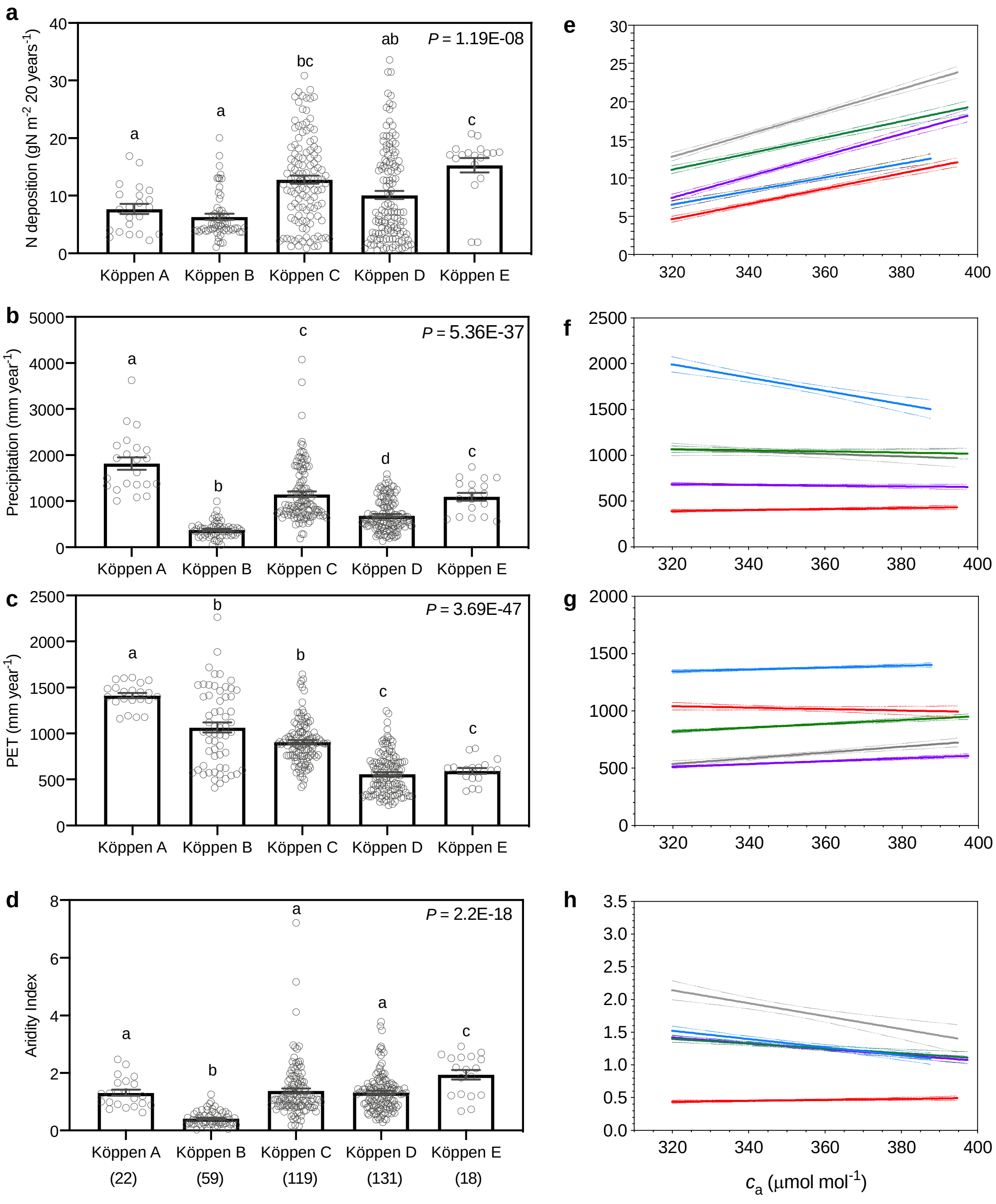

Supplementary Fig. 2. Nitrogen deposition and climate measures for study sites within Köppen Climate zones and relationships to atmospheric CO_2 (c_a, µmol mol^{-1}). Based on global dataset of 10921 individual tree-ring isotope measurements across 349 sites for the period 1965–2015. Panels a and e: cumulative nitrogen deposition over twenty years (N deposition, gN m^{-2} 20 years^{-1}). Panels b and f: precipitation (*P*, mm year^{-1}). Panels c and g: potential evapotranspiration (*PET*, mm year^{-1}). Panels d and h: Aridity Index (annual precipitation/annual potential evapotranspiration). For panels a to d comparisons are from linear mixed models, post-hoc difference at $P < 0.05$. For panels e to h, comparisons are from linear regressions, showing best fit and 95% confidence intervals. P, PET and Aridity Index are based on TerraClimate data. Number of sites in each Köppen Climate zone shown in parentheses.

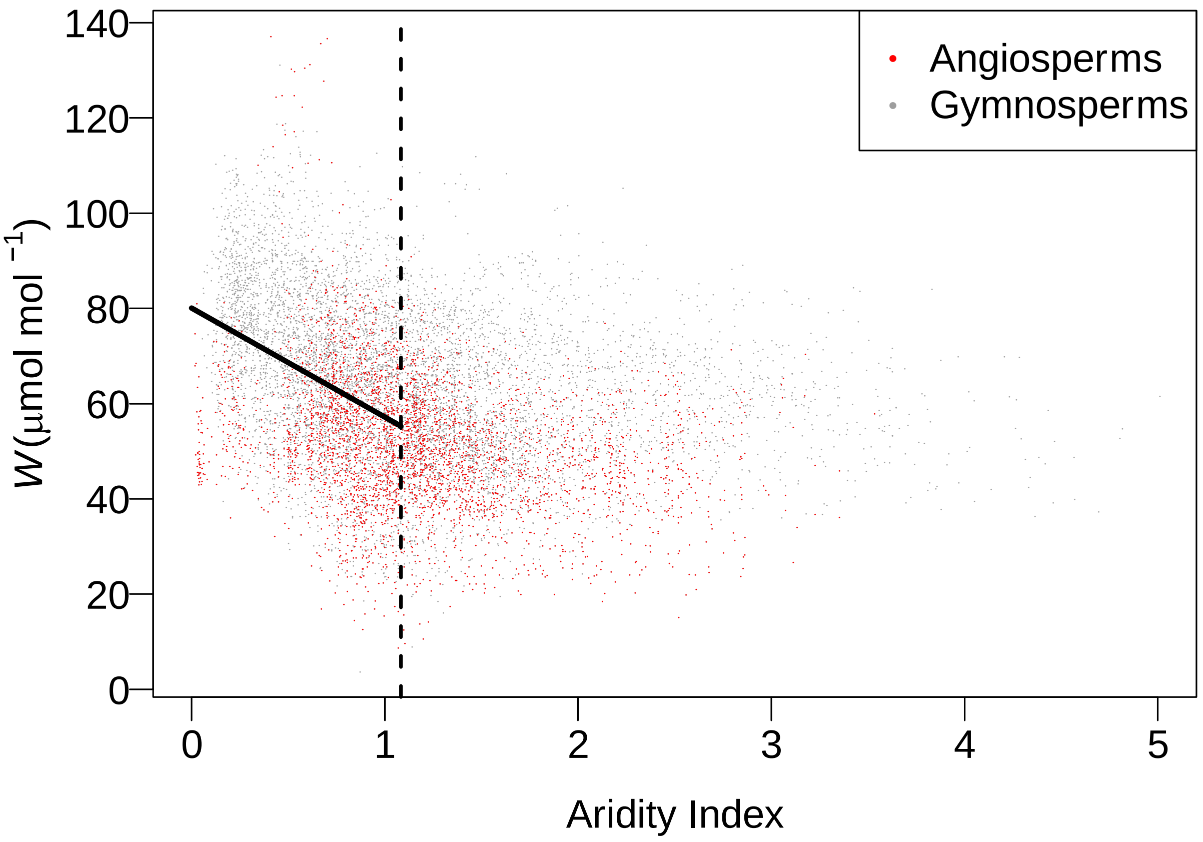

Supplementary Fig. 3 Distribution of Angiosperms and Gymnosperms within the global data (see Fig. 2). Angiosperms are shown in red. The regression line shown is for Angiosperms+Gymnosperms (after Fig. 2). All Angiosperms with *W* > 100 are leguminous.

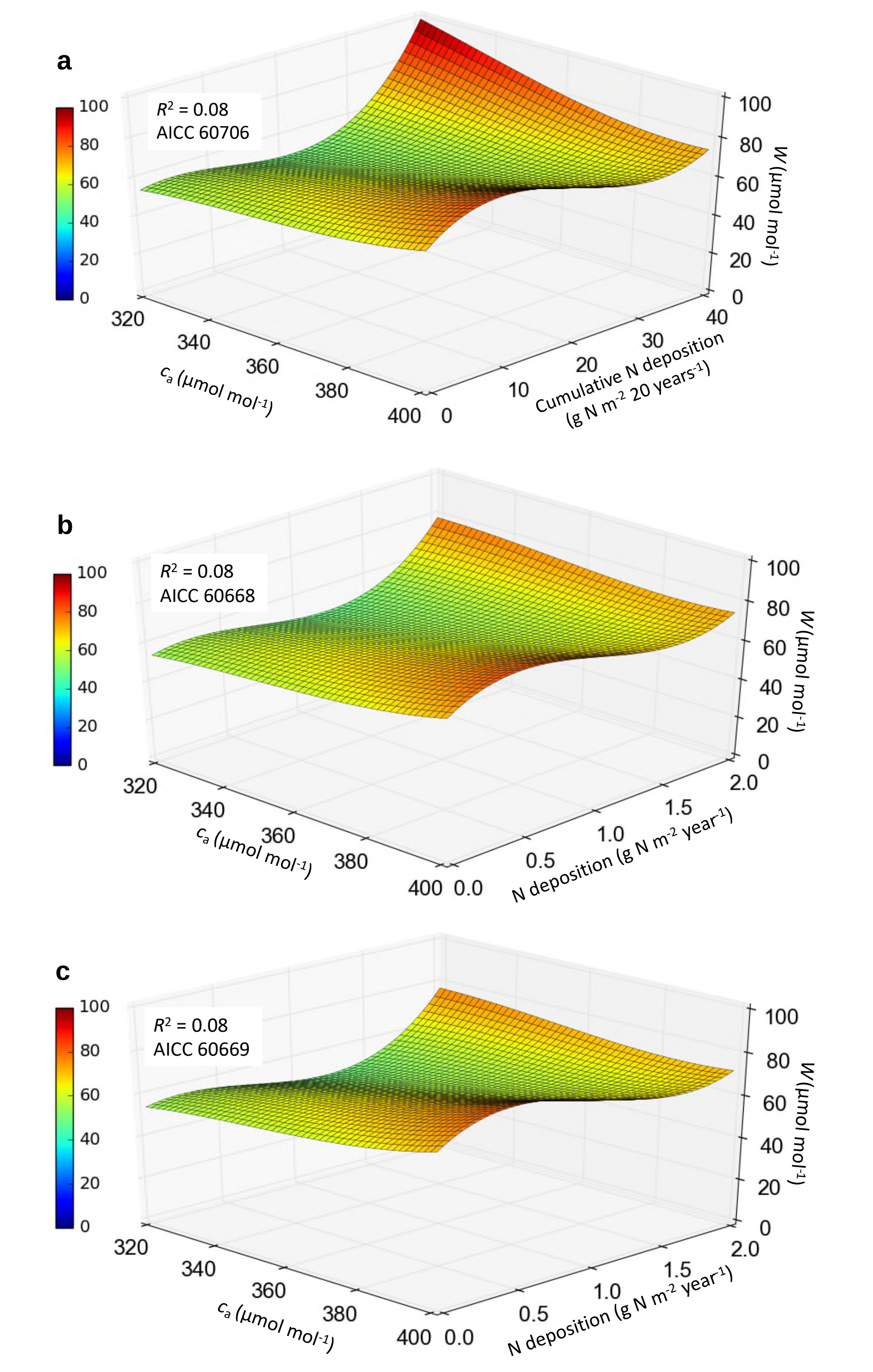

Supplementary Fig. 4 Influence of atmospheric CO_2 (c_a, µmol mol^{-1}) and N deposition (gN m^{-2}) on intrinsic water use efficiency (*W*, µmol mol^{-1}) for the period 1965–2015. Shown are polynomial models of combined effect of c_a and N deposition on *W.* AICC denotes Akaike's Information Criterion. (a) Cumulative N deposition (gN m^{-2} 20 years^{-1}), (b) Annual N deposition (gN m^{-2} year^{-1}), (c) N deposition for previous year (i.e. lagged by one year; gN m^{-2} year^{-1}).

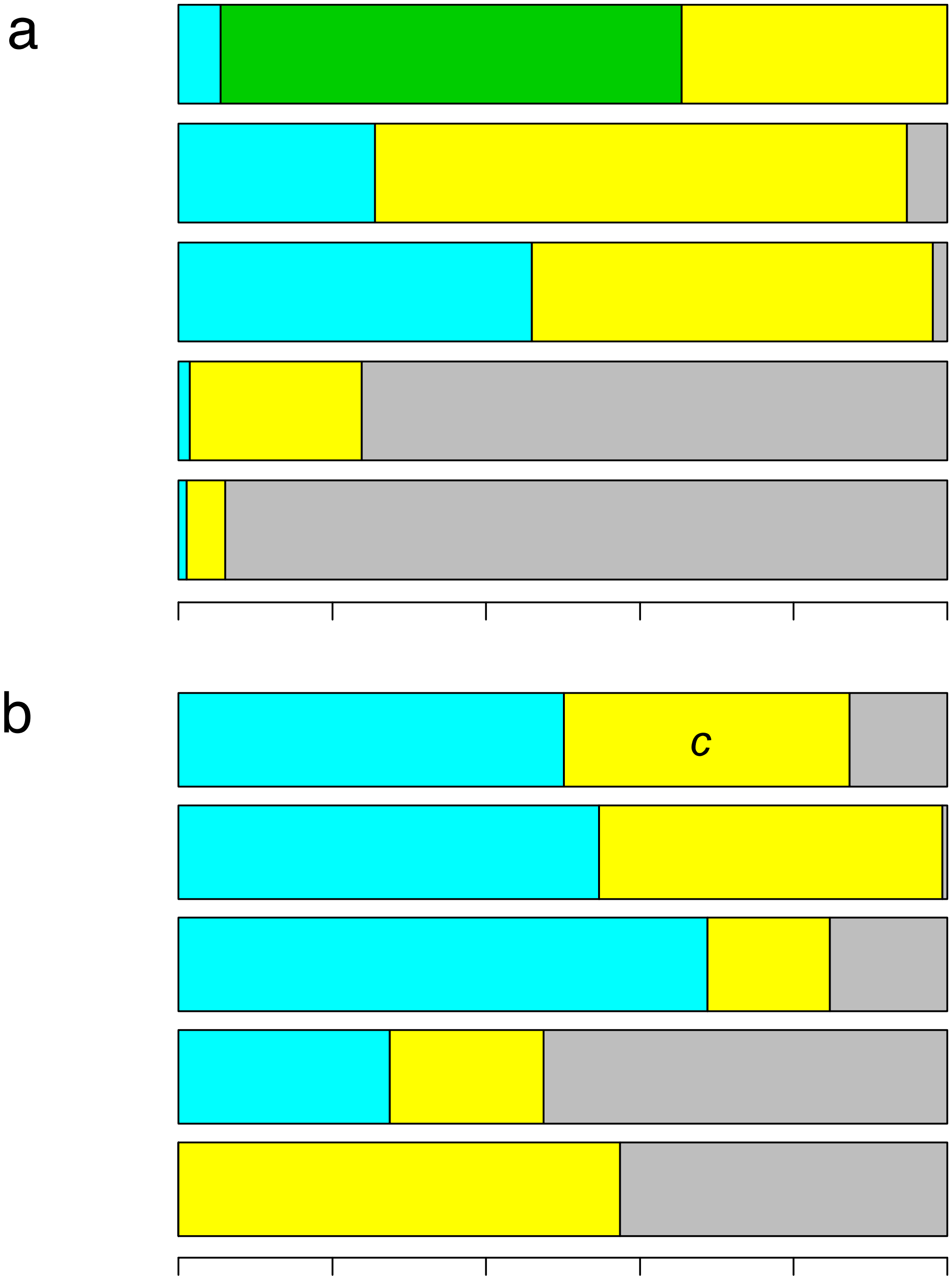

Supplementary Fig. 5. Contributions fixed effect variance (marginal R^2) in multivariate models (Supplementary Table 1) of *W* (µmol mol^{-1}) for the period 1965–2015. Fixed effects were atmospheric CO_2 (c_a, µmol mol^{-1}), Precipitation (mm $year^{-1}$), Potential Evapotranspiration (mm $year^{-1}$) and cumulative nitrogen deposition over twenty years (g N m^{-2} 20 $years^{-1}$. Climatic parameters were derived from (a) TerraClimate database, (b) CRU database.

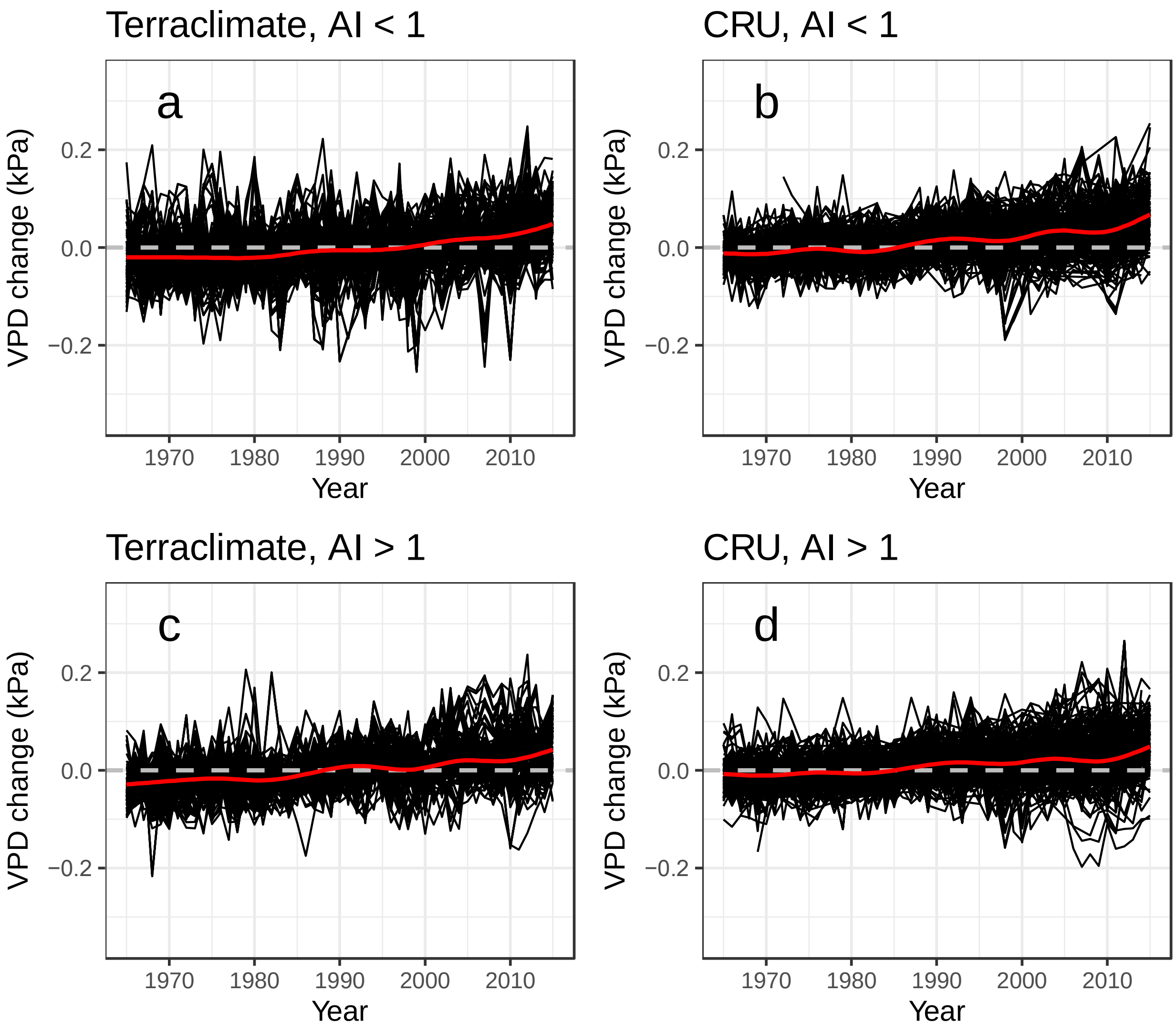

Supplementary Fig. 6. Long-term variation in vapour pressure deficit (VPD). We show the difference (red line, smoothed using generalized additive models) between annual mean VPD and long-term mean VPD for every site. The dashed line indicates differences = 0. Data were parsed according to AI (<1, >1) and data source. Using CRU data, we calculated VPD as the difference between saturation vapour pressure (estimated with the August–Roche–Magnus formula) and air vapour pressure.

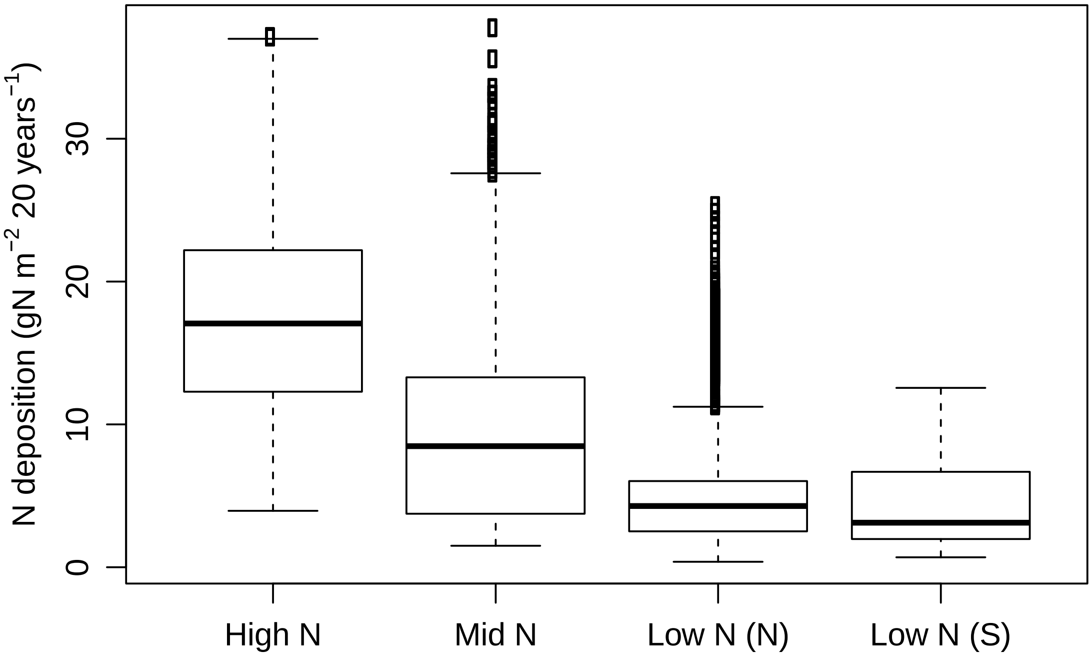

Supplementary Fig. 7 Cumulative N deposition for study sites within N deposition zones (see Methods for definition of N deposition zones). Medians are represented by bold horizontal line, quartiles by the box, and whiskers extend to 1.5 x interquartile range. Observations outside this range are shown as dots. The number of observations (*n*), that is trees for High N region is 4577, for Mid N *n* = 2111, for Low N (N) *n* = 3350 and for Low N (S) *n* = 903. N deposition data is derived from the ISIMIP database (see Methods).

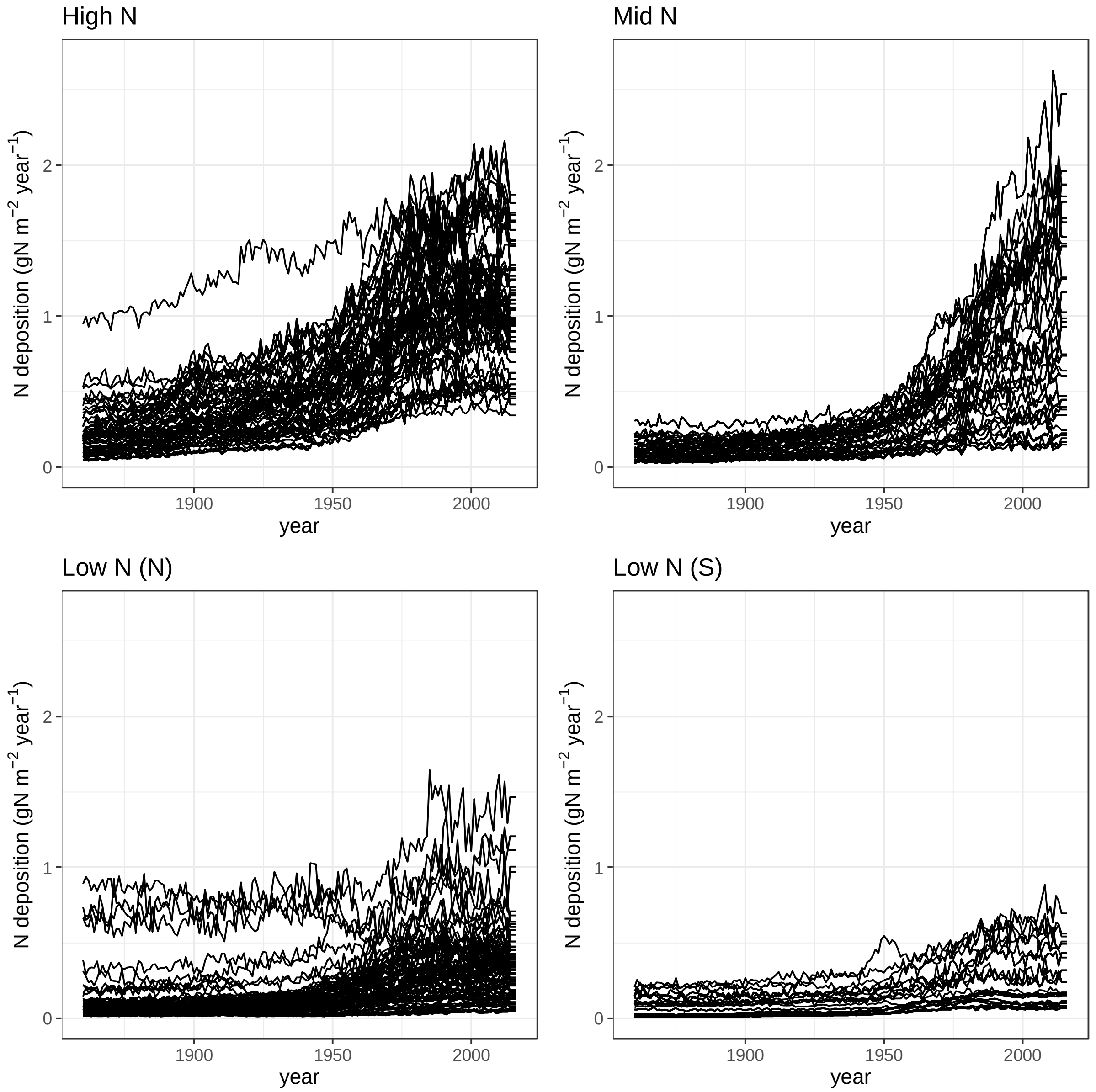

Supplementary Fig. 8 Annual N deposition (based on ISIMIP database) for individual study sites within N deposition zones (see Methods for definition of N deposition zones) for the period 1860-2020. The range shown extends back to 1850.
