# Supplementary material for: CO2, nitrogen deposition and a discontinuous climate response drive water use efficiency in global forests
Source: Nat Commun. 2021 Aug 31;12:5194. doi: 10.1038/s41467-021-25365-1 (PMC8408268; doi:10.1038/s41467-021-25365-1)
Supplement: Supplementary file 2 — Reporting Summary [file 41467_2021_25365_MOESM2_ESM.pdf]

nature portfolio

Corresponding author(s): Mark A. Adams

Last updated by author(s): Jun 28, 2021

Reporting Summary

Nature Portfolio wishes to improve the reproducibility of the work that we publish. This form provides structure for consistency and transparency in reporting. For further information on Nature Portfolio policies, see our Editorial Policies and the Editorial Policy Checklist.

Statistics

For all statistical analyses, confirm that the following items are present in the figure legend, table legend, main text, or Methods section.

n/a	Confirmed	
☐	☒	The exact sample size (n) for each experimental group/condition, given as a discrete number and unit of measurement
☒	☐	A statement on whether measurements were taken from distinct samples or whether the same sample was measured repeatedly
☐	☒	The statistical test(s) used AND whether they are one- or two-sided<br>*Only common tests should be described solely by name; describe more complex techniques in the Methods section.*
☐	☒	A description of all covariates tested
☐	☒	A description of any assumptions or corrections, such as tests of normality and adjustment for multiple comparisons
☐	☒	A full description of the statistical parameters including central tendency (e.g. means) or other basic estimates (e.g. regression coefficient) AND variation (e.g. standard deviation) or associated estimates of uncertainty (e.g. confidence intervals)
☐	☒	For null hypothesis testing, the test statistic (e.g. F, t, r) with confidence intervals, effect sizes, degrees of freedom and P value noted<br>*Give P values as exact values whenever suitable.*
☒	☐	For Bayesian analysis, information on the choice of priors and Markov chain Monte Carlo settings
☒	☐	For hierarchical and complex designs, identification of the appropriate level for tests and full reporting of outcomes
☒	☐	Estimates of effect sizes (e.g. Cohen's d, Pearson's r), indicating how they were calculated

Our web collection on statistics for biologists contains articles on many of the points above.

Software and code

Policy information about availability of computer code

Data collection	No software was used to collect data.
Data analysis	We used R for statistical analysis (see text for full details).

For manuscripts utilizing custom algorithms or software that are central to the research but not yet described in published literature, software must be made available to editors and reviewers. We strongly encourage code deposition in a community repository (e.g. GitHub). See the Nature Portfolio guidelines for submitting code & software for further information.

Data

Policy information about availability of data

All manuscripts must include a data availability statement. This statement should provide the following information, where applicable:

- Accession codes, unique identifiers, or web links for publicly available datasets
- A description of any restrictions on data availability
- For clinical datasets or third party data, please ensure that the statement adheres to our policy

All data were sourced from publicly available repositories. Our compiled tree ring data are available at https://doi.org/10.1038/s41558-020-0747-7. The nitrogen deposition data are available at: https://www.isimip.org. We used the Terraclimate and CRU data bases for climate data and full details (including links) are available in the text. We have described the sources of the CO2 data and provided full citations to those sources.

Field-specific reporting

Please select the one below that is the best fit for your research. If you are not sure, read the appropriate sections before making your selection.

☐ Life sciences ☐ Behavioural & social sciences ☒ Ecological, evolutionary & environmental sciences

For a reference copy of the document with all sections, see nature.com/documents/nr-reporting-summary-flat.pdf

Ecological, evolutionary & environmental sciences study design

All studies must disclose on these points even when the disclosure is negative.

Study description	We tested the significance (using multivariate mixed models) of different drivers of water use efficiency in forests and woodlands across the globe.
Research sample	As noted above (Data), we used a data base for carbon isotope composition of tree ring wood that we previously compiled (See Adams et al 2020 for details). We combined this with with publicly available data for atmospheric CO2 (details provided in the text) and for climate (see Data, above) and nitrogen deposition (see Data, above).
Sampling strategy	The data sets chosen for this study are the most complete at this time. The tree-ring data base we compiled is unique, while the Terraclimate and CRU data bases for climate are widely regarded as the best-available and have been widely used in similar studies. Nitrogen deposition data were taken from the ISIMP project (available via the ISIMP website). The ISIMP data are regarded as the most authoritative source for nitrogen deposition at the spatial and temporal scales used in our study.
Data collection	We provide citations for each of the data sets used in our study. Those citations detail who collected / created the data, and how the data were compiled, including methods used for any required corrections.
Timing and spatial scale	The data used here covers the period 1965-2015. We used annual data, corresponding to tree-ring ages, as this provided the most appropriate measurement unit (tree rings are mostly annual). The duration of the study was determined on the basis of availability of nitrogen deposition data.
Data exclusions	All analyses were based on the full data set for tree rings (as reported by Adams et al 2020). In one analysis (see Table 1) we excluded data for extremes of Aridity Index. That exclusion is noted in full in the text. We also note the effect of that exclusion on the results.
Reproducibility	We did not use experimental data and this field is not applicable to the present study as there are no alternate sources of data for tree ring isotopes.
Randomization	Data were treated as continuous, unless otherwise noted. Nitrogen deposition data were classified geographically (latitude, longitude) into three broad zones, as described in the text. Randomization was not applicable owing to the nature of our study.
Blinding	Blinding was not required as our study did not involve subjective anaslysis.

Did the study involve field work? ☐ Yes ☒ No

Reporting for specific materials, systems and methods

We require information from authors about some types of materials, experimental systems and methods used in many studies. Here, indicate whether each material, system or method listed is relevant to your study. If you are not sure if a list item applies to your research, read the appropriate section before selecting a response.

Materials & experimental systems

n/a	Involved in the study
☒	☐ Antibodies
☒	☐ Eukaryotic cell lines
☒	☐ Palaeontology and archaeology
☒	☐ Animals and other organisms
☒	☐ Human research participants
☒	☐ Clinical data
☒	☐ Dual use research of concern

Methods

n/a	Involved in the study
☒	☐ ChIP-seq
☒	☐ Flow cytometry
☒	☐ MRI-based neuroimaging
